# Within-player load-wellness monitoring and lower-limb pain in elite female football: A reproducible secondary analysis of the SoccerMon dataset

**DOI:** 10.1371/journal.pone.0362663

**Published:** 2026-11-13

**Authors:** Yushuo Liu, Haitao Li

**Affiliations:** 1 School of Physical Education, Yunnan Normal University, Kunming, Yunnan, China; 2 Department of Rehabilitation Medicine, Cangzhou Second Hospital, Cangzhou, Hebei, China; Shahid Beheshti University, IRAN, ISLAMIC REPUBLIC OF

## Abstract

Routine athlete-monitoring data can test whether within-player changes in training load and wellness precede health problems, but sparse outcomes and incomplete reporting complicate inference. We conducted a reproducible secondary longitudinal analysis of the public SoccerMon dataset, comprising 50 players from two Norwegian elite women’s football teams monitored in 2020–2021. The outcome was onset on day t (the index player-day) of a self-reported lower-limb pain/injury episode after a 7-day episode washout. The primary risk set contained 42 onset-days from 15 players; the complete-case soreness interaction analysis contained 28 onset-days from nine event players. Under retrospective full-period within-player standardization, the focal acute-load x soreness interaction was estimated below 1.0 by Firth logistic regression (odds ratio [OR] 0.63, 95% confidence interval [CI] 0.45–0.97), but uncertainty remained substantial. When within-player standardization was recalculated using only observations available before each day, the interaction attenuated to OR 0.97 (0.73–1.25). These data therefore do not provide stable evidence that within-player load deviations or load x wellness interactions are associated with next-day self-reported lower-limb pain onset. Sparse events, event concentration, missing monitoring data, and team/season imbalance further limit precision. In practice, daily load and wellness signals should be interpreted as contextual longitudinal information rather than as stand-alone next-day risk thresholds.

## Introduction

Daily athlete monitoring combines measures of training exposure, perceived exertion, recovery, and wellness to support training decisions. Subjective measures can respond quickly to training stress and are widely used because they are inexpensive and feasible in routine practice [1,2]. In football, however, a monitoring value is rarely interpreted in isolation: staff typically compare a player’s current value with her own recent or usual level. This within-player perspective is clinically and operationally attractive, but it also creates statistical challenges because the same player contributes repeated observations, exposure windows overlap, and reporting is incomplete.

The evidence base is particularly limited in women’s football. A systematic review of training-load monitoring in highly trained and elite adult women players found only 16 eligible studies and concluded that dose-response relations between training load, fatigue, adaptation, and injury remain insufficiently characterized [3]. A broader 2025 review likewise emphasized that integrated assessment of external load, internal load, and well-being remains heterogeneous across football settings [4]. Injury-surveillance syntheses show that lower-limb injuries account for a large share of the burden in women’s football, while estimates vary with competition level, exposure definition, and injury definition [5,6]. Reviews of prospective risk factors in female field and court sports also show that previous injury is among the more reproducible markers, whereas many proposed screening variables have uncertain or context-dependent associations [7]. Exercise-based injury-prevention programmes can reduce injury incidence in women’s football, but monitoring-based risk thresholds have not been established with comparable evidence [8].

Recent football studies further illustrate why load and health cannot be reduced to a single universal threshold. Functional movement characteristics in elite female youth football are related to performance qualities such as speed, agility, and jump capacity, but such cross-sectional associations do not establish prospective injury-risk cut-points [9]. Training-design studies in youth football show that small-sided games, dribbling circuits, and different agility formats can produce distinct physical, technical, internal-load, and enjoyment responses [10,11]. These studies are not injury-risk studies; rather, they reinforce that a given internal-load value depends on training content, athlete characteristics, and context. For routine monitoring, the relevant question is therefore not whether one absolute load is ‘safe’ or ‘unsafe,’ but whether deviations from an individual’s own pattern contain reproducible information about subsequent symptoms.

The public SoccerMon resource offers a rare opportunity to examine this question in elite women’s football because it links daily wellness, session rating of perceived exertion (sRPE), and self-reported injury/pain records from two Norwegian elite teams across two seasons [12,13]. The present study is a secondary longitudinal analysis of those subjective monitoring data. The analysis was originally registered on the Open Science Framework (OSF) using onset on day t, where t denotes the current/index player-day, strictly lagged exposures, and wellness variables coded so that higher transformed values consistently represented a worse state [14]. During reanalysis, the previously reversed fatigue interpretation was corrected, recurrent-event risk sets were reconstructed, and a frozen revision-stage statistical analysis plan (SAP) was implemented. Subsequent sensitivity analyses addressed exposure construction, team and season instability, and model-space multiplicity; these are distinguished from both the original registration and the frozen revision-stage SAP.

A transient increase in training demand may have different implications depending on a player’s recovery state. Greater-than-usual soreness may reflect recent musculoskeletal loading, incomplete recovery, or symptom perception, whereas fatigue and readiness may capture broader recovery status. On this basis, we expected any association between higher-than-usual acute load and next-day symptom onset to be more pronounced when wellness was worse than usual.

Our primary scientific hypothesis was that higher-than-usual acute training load would be associated with greater next-day odds of a new self-reported lower-limb pain/injury episode when wellness was worse than usual, with acute load x soreness burden designated as the focal interaction. Fatigue burden and readiness deficit were prespecified alternative wellness variables. We did not aim to construct a prediction tool, estimate a clinically actionable threshold, or compare women with men. The novelty is instead methodological and substantive: to test a registered within-player load-wellness hypothesis in an open elite-women dataset while making the consequences of recurrent events, scale direction, missing reporting, sparse events, and alternative exposure constructions directly auditable.

## Methods

### Study design, registration, and analysis hierarchy

This study is a retrospective secondary longitudinal analysis of prospectively collected athlete-monitoring data. The unit of analysis is the player-day. Reporting follows STROBE and, where applicable, the RECORD extension for routinely collected data [15,16] (S1 Text). The unchanged OSF preregistration is cited in [14] (S3 File). The revision-stage SAP, frozen on 22 July 2026 before inferential refitting, designated a player-random-intercept logistic mixed model as the intended primary estimator and Firth regression as a small-sample companion. Its historical input hash and model-decision records are retained in S2 File. Later sensitivities – non-overlapping chronic load, team/season removal, backward-looking standardization, and a model-space census – were post-SAP rather than originally prespecified. S10 Table reconciles the original registration, frozen SAP, and final analytical prioritization.

### Data source, participants, and data collection

We accessed the public SoccerMon dataset from Zenodo (DOI 10.5281/zenodo.10033832) and used the accompanying Scientific Data descriptor [12,13]. SoccerMon contains data from two teams in the Norwegian elite women’s league during the 2020 and 2021 seasons. Subjective data were collected with the PmSys athlete-monitoring system: players submitted session-level training-load reports, daily wellness reports, and injury reports through a smartphone application. Training load included session duration and rating of perceived exertion; wellness items included fatigue, readiness, soreness, mood, stress, sleep quality, and sleep duration; injury reports recorded body location and severity [12]. Descriptive training/session and wellness summaries are provided in S1 Table and S2 Table.

The subjective release used in this analysis contains 50 anonymized players (Team A, 27; Team B, 23). Because the public dataset removes player metadata, age, height, body mass, playing position, and other participant-level demographic characteristics are not available and were not inferred or imputed. Accordingly, the reproducible participant description is limited to sex, elite competition level, team, season, and anonymized player identifier. The final analysis used a common study grid spanning 1 January 2020 to 31 December 2021 (731 days per player; 36,550 theoretical player-days). The preregistration instead defined each player’s observation period from the first to the last valid subjective monitoring record. The common calendar grid was retained because daily load is provided on each calendar day and the risk set is defined in calendar time; days outside a player’s reporting window contain no wellness report and therefore do not enter wellness complete-case models. This deviation from the registered observation-period definition is documented in S10 Table.

The dataset descriptor reports written informed consent for participation and open publication of anonymized data, approval by the Norwegian Privacy Data Protection Authority (reference 296155), and exemption from review by the relevant Regional Committees for Medical and Health Research Ethics [12]. The descriptor does not provide an explicit Declaration of Helsinki statement; we therefore do not claim one. No new participant contact, intervention, or identifiable information was used in this secondary analysis, and no additional ethics approval was required.

### Raw-data integrity and polarity verification

All analysis variables were rebuilt from the public raw files. File-level SHA-256 hashes were recorded, and an independent raw-data rebuild was reconciled against the reference analytical dataset; all predefined data-integrity checks passed. A concise code-reproduction record is provided in S1 File, and the complete reproducibility materials are archived in S2 File. Self-reported injury/pain records were expanded to one row per body location before episode construction.

Wellness polarity was verified independently of the outcome. The published PmSys screenshot establishes that higher raw fatigue scores indicate greater freshness and that higher readiness scores indicate greater readiness [12]. For soreness, the same PmSys instrument is documented independently in Jarmann’s 2023 thesis with anchors ranging from 1 = ’Very sore’ to 5 = ’Feeling great’ [17]. The polarity decision was made without using onset status, exposure-outcome associations, model coefficients, p values, or outcome correlations. We therefore defined fatigue_burden = 6 – fatigue, soreness_burden = 6 – soreness, and readiness_deficit = 11 – readiness, so that higher transformed values consistently represent a worse-than-usual state.

### Training load, episodes, and risk set

Daily training load was derived from session sRPE, defined as session rating of perceived exertion multiplied by session duration [18]. Acute load was the mean daily load over days t-7 to t-1 and chronic load the mean over days t-28 to t-1. The dataset-provided 28-day chronic training load (CTL28) field was used only for descriptive validation and was not the modeled chronic exposure.

Injury/pain reports were converted to episodes separately by player and exact body location, distinguishing left and right sides. Reports separated by no more than 7 days were merged into the same episode; the first date defined episode onset. Three- and 14-day washouts were retained as sensitivity analyses (S6 Table). The lower-limb region comprised hip/groin, thigh, knee, lower leg, ankle, and foot. The primary outcome was a binary indicator of new onset of any lower-limb episode on day t. Multiple locations beginning on the same player-day were collapsed to one onset-day. Days during an ongoing episode, beginning the day after onset and continuing through the last report in that episode, were excluded from the risk set; players re-entered the following day. A stricter re-entry sensitivity required an additional washout-length gap. The SoccerMon injury records are subjective reports of location and severity and do not include clinical diagnoses [12]. Thus, the study outcome represents a self-reported symptom episode rather than a clinician-confirmed diagnosis; self-reported health-problem surveillance is widely used in sport monitoring [19].

### Primary and alternative exposure parameterization

Wellness exposures used the most recent available report within days t-3 to t-1, consistent with the registered recent-window rule; strict t-1 reporting was retained as a sensitivity analysis. In the revision-stage reference analysis, load and wellness summaries were converted to within-player z-scores using each player’s mean and standard deviation over the full available observation period, requiring at least 10 non-missing observations and non-zero standard deviation. This parameterization is retrospective: it describes a day relative to the player’s overall observed distribution and is not available prospectively in real time.

Two additional exposure sensitivities were evaluated without replacing the reference estimand. First, to separate the mathematically overlapping load windows, chronic load was redefined as the mean over t-28 to t-8 while acute load remained t-7 to t-1. Second, backward-looking standardization used only prior exposure-summary observations before day t to compute each player’s expanding-history mean and sample standard deviation, requiring at least 10 prior non-missing observations and non-zero standard deviation; day t and all future observations were excluded. Because the preregistration did not specify a fixed-duration baseline, the reported sensitivity used an expanding-history baseline to eliminate future-data leakage without introducing an additional post hoc fixed-window choice.

### Statistical analysis

The registered focal contrast was the acute-load deviation x soreness-burden deviation. The original OSF preregistration planned a joint main-effects model including soreness, fatigue, and readiness, with acute load x soreness as the focal interaction and fatigue/readiness as alternative interaction variables. The frozen revision-stage SAP specified separate wellness-variant model families, with a player-random-intercept discrete-time logistic mixed model as the intended primary estimator and Firth penalized logistic regression as a small-sample companion. The final focal soreness analysis used complete-case player-days and retrospective full-period within-player standardization. Because Team B had no complete-case onset and only 28 onset-days occurred in nine event players, final interpretation prioritized Firth estimates and whole-player bootstrap uncertainty rather than the separated, unpenalized population-average fallback. Diagnostics and full coefficients are reported in S4 and S5 Tables, respectively.

For the focal soreness complete-case analysis (19,758 at-risk player-days from 49 players), the exact Firth specification was: onset_day ~ acute_load_7d_wp_z * soreness_burden_recent3_wp_z + chronic_load_28d_wp_z + prev_episode_ever + calendar_time + team + season. The asterisk denotes the two main effects and their interaction; team and season were categorical factors. Firth estimates, profile-likelihood 95% confidence intervals, and p values were obtained using R’s logistf package. The Firth model did not contain a player random intercept or cluster-robust standard errors. To examine within-player dependence separately, 2,000 bootstrap samples resampled whole players and refitted the same Firth fixed-effects specification using independently validated Firth IRLS code; the resulting percentile intervals are shown separately from the model-based intervals (Table 2; S8 Table).

Key robustness analyses examined backward-looking standardization, a non-overlapping chronic-load window, and team/season instability (S7 Table). Alternative wellness models, recurrent-event estimators, episode-definition and risk-set sensitivities, acute:chronic workload ratio analyses, strict t-1 wellness, and influence analyses are reported in the Supporting Information. Washout results are summarized in S6 Table and bootstrap/cluster-robust inference in S8 Table [20–22].

Missing wellness data were handled by model-specific complete-case analysis and inverse-probability-of-observation weighting (IPW) as a sensitivity analysis [23]. Stabilized weights were truncated at the 1st and 99th percentiles and weighted logistic models used player-cluster small-sample bias-reduced cluster-robust (CR2) inference; an alternative observation model omitted onset status. Detailed availability, positivity, weight-distribution, and lag-alignment diagnostics are provided in S9 Table. Because the weighting procedure remained dependent on strong modeling assumptions and retained residual alignment uncertainty, IPW was not interpreted as resolving missing-data or selection bias. The preregistration also planned a conservative sensitivity analysis treating unreported training-load days as missing rather than zero when a rest-day interpretation was unsupported. This analysis was not executable because SoccerMon provides a daily-load value on every calendar day, with zero on days without a recorded session, so true rest days cannot be distinguished from unreported training days; this deviation is documented in S10 Table.

Additional diagnostics included Pearson and Spearman correlations and variance inflation factors (VIFs) for acute and chronic load, the non-overlapping t-28 to t-8 chronic-load analysis, refits with team or season removed, and the backward-looking standardization described above (S3 Table and S7 Table). Sparse-data diagnostics recorded event counts, event players, parameters, crude events-per-parameter, separation, aliasing, and model failures (S4 Table). No stepwise selection or p-value-driven model reduction was used.

Finally, every fitted specification and focal coefficient test was recorded in the model registry to place isolated nominal p values in context. For multiplicity reporting, the scientifically reported model space was defined as successfully fitted primary, key-sensitivity, and secondary specifications that contributed to scientific interpretation; internal quality-assurance fits, failed fits, and rejected fallback levels were excluded from this denominator and retained in the reproducibility archive. No Bonferroni or false-discovery-rate procedure was introduced as a new primary inferential framework because these overlapping analyses do not constitute one homogeneous family of independent confirmatory tests. Effect estimates and 95% confidence intervals are emphasized, and absence of a stable association is not interpreted as evidence of equivalence or no effect.

Analyses used R 4.6.1 (including survival, lme4, clubSandwich, sandwich, logistf, boot, and coxme) and Python 3.11. The final reproducibility audit reproduced the reference analysis from both derived and raw inputs and passed all predefined validation checks. The code-reproduction record is provided in S1 File; code, logs, structured results, analysis taxonomy, model registry, and hashes are provided in S2 File.

## Results

### Data structure, event concentration, and missingness

The dataset comprised 36,550 theoretical player-days. The 7-day episode construction yielded 92 lower-limb body-location episodes and 52 unique lower-limb onset-days. Ten onset-days occurred during another ongoing episode and were outside the risk set, leaving 42 onset-days from 15 event players in 36,296 at-risk player-days (Fig 1).

**Fig 1:**
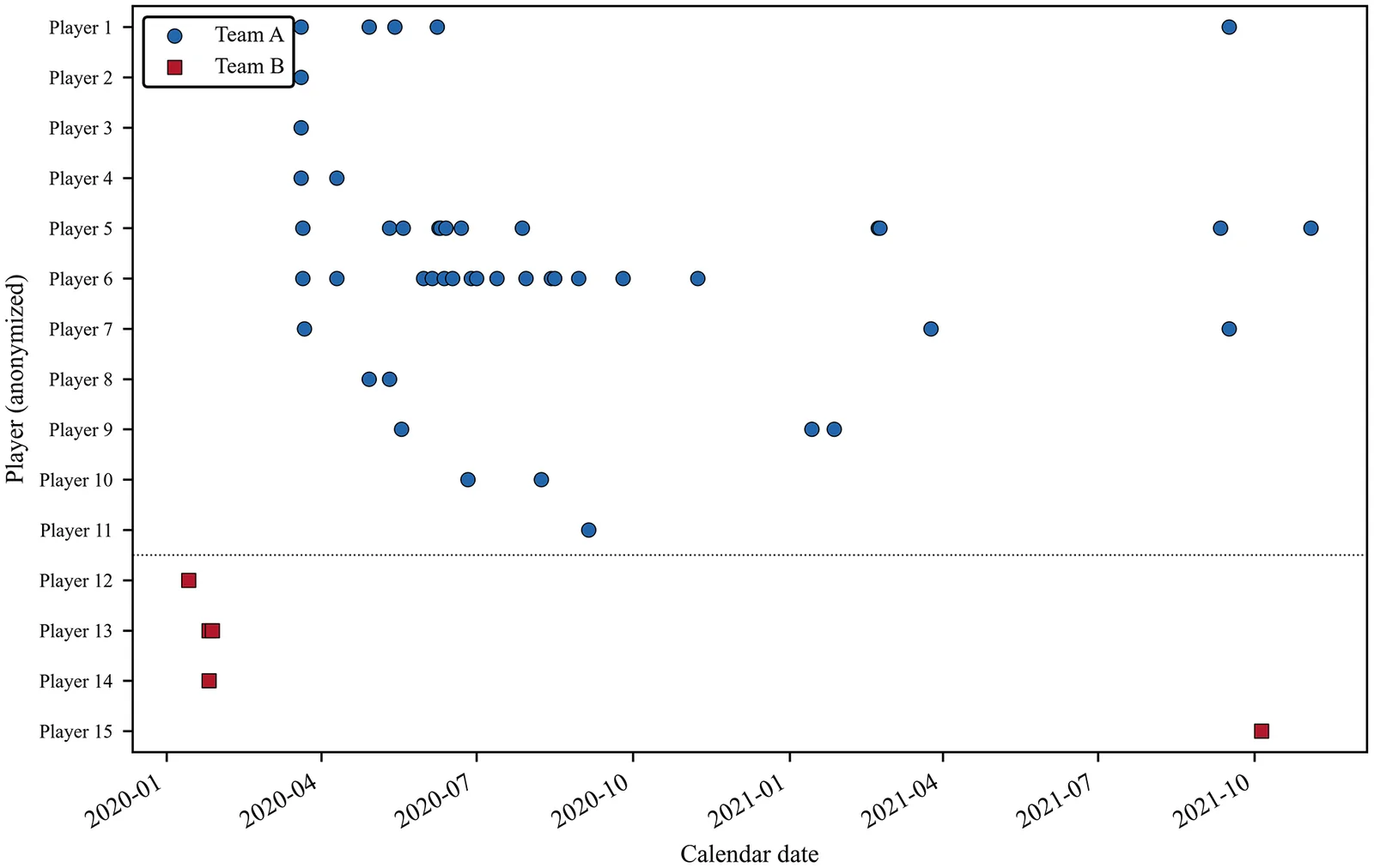
Calendar-time distribution of lower-limb onset-days by team. Each point represents one of 52 unique onset-days under the 7-day washout; same-day multi-location onsets were merged.

Event information was highly concentrated. One player contributed 45 of 92 body-location episodes (48.9%). After same-day locations were collapsed to the player-day onset outcome used for inference, the largest contribution was 11 of 42 risk-set onset-days (26.2%); in the soreness complete-case sample, two players each contributed 6 of 28 onset-days (21.4%; Fig 2).

**Fig 2:**
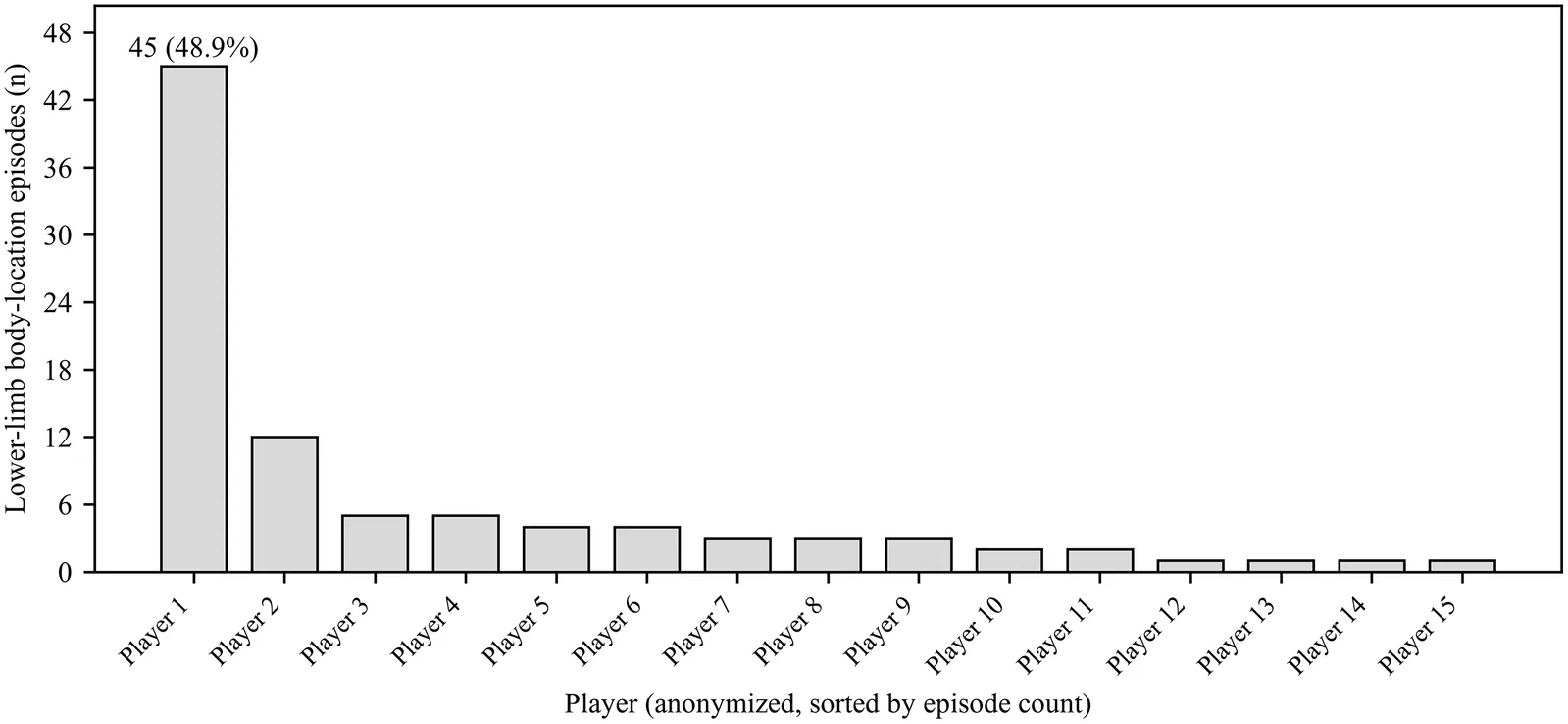
Concentration of lower-limb episodes among event players. Bars show body-location episode counts for the 15 players with at least one episode under the 7-day washout; players are anonymized and sorted.

The soreness complete-case model included 19,758 at-risk player-days from 49 players, with 28 onset-days from nine event players. Fatigue and readiness models included 19,815 and 19,817 player-days, respectively, and the same 28 onset-days from nine event players. Thus, the complete-case models retained about 54% of the full risk-set days and two-thirds of its onset-days. In the soreness complete-case sample, all 28 onset-days occurred in Team A; Team B contributed 7,256 player-days but no event. Season was also imbalanced: 20 of 28 onset-days (71.4%) occurred in 2020 and 8 (28.6%) in 2021 (Table 1).

**Table 1:** Data structure, event distribution, and monitoring availability.

Quantity	Value
Players (Team A / Team B)	50 (27 / 23)
Observation period	2020-01-01 to 2021-12-31
Theoretical player-days	36,550
Primary at-risk player-days	36,296
Lower-limb episodes, 7-day washout	92
Unique lower-limb onset-days	52
Onset-days in primary risk set	42 from 15 event players
Soreness complete-case sample	19,758 days; 28 onsets; 9 event players
Fatigue complete-case sample	19,815 days; 28 onsets; 9 event players
Readiness complete-case sample	19,817 days; 28 onsets; 9 event players
Soreness CC events by team	Team A 28; Team B 0
Soreness CC events by season	2020: 20; 2021: 8
Highest risk-set onset-day contributor	11/42 (26.2%)

Notes. CC, complete case. Episode counts are body-location episodes; onset-days collapse multiple same-day lower-limb locations within a player.

### Primary main effects and focal soreness interaction

In the population-average fallback model, acute-load deviation was imprecisely estimated (OR 0.70, 95% CI 0.30–1.64), as were soreness burden (OR 0.74, 0.44–1.26) and chronic-load deviation (OR 0.95, 0.53–1.71). Previous episode history had the largest estimated association (OR 6.49, 1.66–25.35), but its interval was wide. The Team B coefficient was not interpretable because of complete separation, and the season estimate was also unstable (2021 vs 2020 OR 2.69, 0.36–20.17).

Under the retrospective full-period within-player standardization, the focal acute-load x soreness interaction was estimated below 1.0 by Firth logistic regression (OR 0.63, 95% CI 0.45–0.97), but whole-player bootstrap intervals included 1.0. The cross-estimator interaction summary is shown in S1 Fig, with bootstrap details in S8 Table. IPW and alternative-wellness estimates are reported in Table 2 and S9 Table and are interpreted as sensitivity analyses rather than independent replication (Table 2; Fig 3).

**Table 2:** Reference main-effect estimates and prioritized focal interaction estimates.

Analysis / term	Effect ratio	95% CI	p
Population-average fallback: acute load	0.696	0.296-1.637	0.407
Population-average fallback: soreness burden	0.745	0.440-1.260	0.272
Population-average fallback: chronic load	0.953	0.532-1.707	0.873
Population-average fallback: previous episode history	6.486	1.659-25.354	0.007
Firth acute x soreness	0.630	0.445-0.965	0.035
Firth + player-cluster bootstrap	0.655	0.440-1.159	–
Marginal player-cluster bootstrap	0.670	0.447-1.107	–
IPW, observation model includes onset	0.646	0.429-0.973	0.037
IPW, observation model omits onset	0.657	0.425-1.015	0.059

Notes. The unpenalized population-average estimates are shown descriptively and are not treated as confirmatory because of sparse events and Team B separation; that fallback model does not contain the interaction. Focal interaction inference emphasizes Firth and player-cluster bootstrap results. Firth intervals use profile likelihood without cluster adjustment; the Firth-bootstrap row uses percentile limits from 2,000 whole-player resamples without a p value.

**Fig 3:**
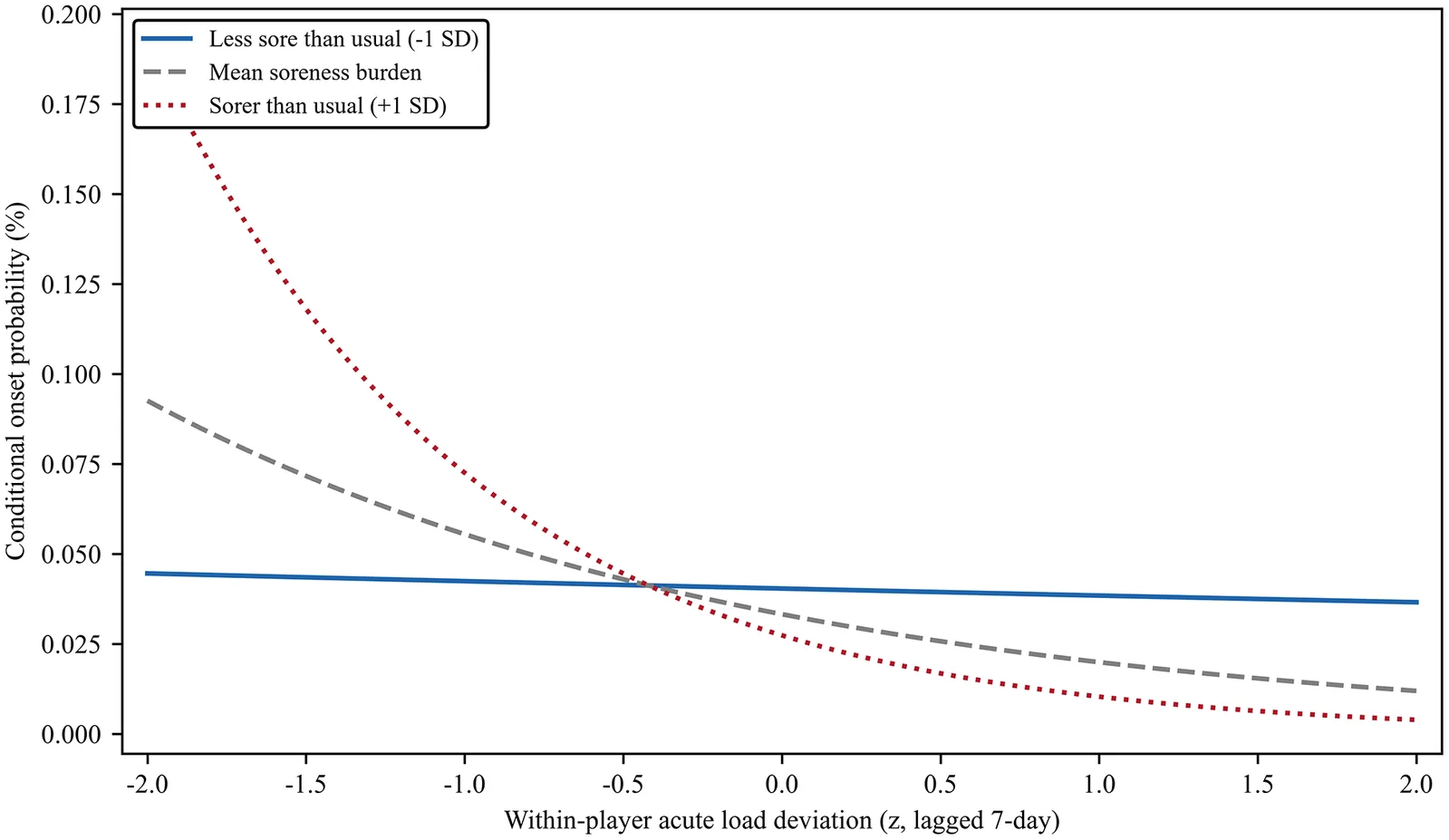
Acute load x soreness-burden interaction under retrospective full-period within-player standardization. Curves are descriptive conditional predictions from the Firth model and illustrate the retrospective estimand; the backward-looking standardization attenuated the interaction toward 1.0 and is emphasized in interpretation.

### Sensitivity analyses of exposure construction and model stability

The acute and chronic within-player load deviations were moderately correlated in the soreness complete-case sample (Pearson r = 0.698; Spearman results were similar). The VIF based on the two load predictors was 1.95; the maximum VIF in the full main-effects design was 4.08 and the scaled condition number was 3.79 (S3 Table). These diagnostics indicate appreciable structural overlap but not extreme multicollinearity. Replacing chronic load t-28 to t-1 with the non-overlapping t-28 to t-8 mean reduced the acute-chronic correlation to r = 0.470. The Firth interaction was essentially unchanged (OR 0.63, 0.45–0.97), and the drop-team mixed model gave OR 0.70 (0.48–1.04) (S7 Table).

Removing team directly addressed complete separation. In like-for-like Firth comparisons, the direction of all focal coefficients was retained: acute load OR 0.60 to 0.71, soreness burden 0.82 to 0.85, chronic load 0.95 to 0.75, previous-episode history 6.13 to 7.10, and the acute-load x soreness interaction 0.63 to 0.67. Confidence intervals remained wide and overlapping; for the interaction, the drop-team Firth interval widened to 0.47–1.07. The full mixed model became estimable after team removal and yielded an interaction OR of 0.69 (95% CI 0.47–1.02). Thus, deleting team did not reverse the focal associations but reinforced their imprecision. Removing season alone also left the Firth interaction essentially unchanged (OR 0.63, 0.44–0.97) (S7 Table).

The backward-looking standardization produced the most important sensitivity result. Requiring at least 10 prior observations reduced the soreness complete-case sample from 19,758 days and 28 onsets to 19,216 days and 27 onsets. Under this temporally implementable scaling, the Firth acute-load x soreness interaction attenuated to OR 0.97 (0.73–1.25). With team removed, the mixed-model and Firth interaction estimates were OR 0.96 (0.70–1.32) and OR 0.94 (0.86–1.14), respectively. At the same time, the chronic-load estimate changed from approximately null under full-period standardization to OR 1.96 (1.21–3.06) in the Firth model and OR 1.94 (1.16–3.24) in the drop-team mixed model. Fatigue and readiness interaction estimates under backward-looking scaling likewise showed no inverse signal. The focal inference therefore depends materially on how the within-player reference distribution is constructed (Table 3; S7 Table).

**Table 3:** Exposure-construction diagnostics and targeted sensitivity analyses.

Question	Key result	Interpretation
Acute/chronic overlap	Pearson r = 0.698; two-predictor VIF = 1.95; max design VIF = 4.08	Moderate structural overlap, not extreme collinearity
Non-overlapping chronic t-28 to t-8	r=0.470; Firth interaction OR 0.630 (0.445-0.966)	Decoupling windows did not materially change interaction
Drop team	Mixed-model interaction OR 0.694 (0.470–1.025)	Separation resolved; inverse estimate remained imprecise
Drop season	Firth interaction OR 0.630 (0.444–0.970)	Season imbalance did not explain the full-period interaction
Backward-looking standardization	Firth interaction OR 0.974 (0.728–1.252); chronic OR 1.955 (1.208–3.058)	Material change in focal interaction and chronic-load estimate
Backward-looking + drop team	Mixed-model interaction OR 0.958 (0.697–1.317)	No inverse interaction signal under prospective-style scaling
Season distribution, soreness CC	2020: 20/28 events; 2021: 8/28 events	Sparse seasonal categories contribute instability
Team distribution, soreness CC	Team A: 28/28 events; Team B: 0/28	Complete separation for team

Notes. These additional sensitivity analyses were not part of the original preregistration. The backward-looking standardization uses only prior observations before day t and requires at least 10 prior non-missing values.

### Missing-data diagnostics and multiplicity

IPW diagnostics remained cautionary. For the soreness model, 19,758 of 36,296 risk-set rows were complete for the modeled data, and the stabilized-weight observation model had an area under the receiver-operating-characteristic curve (AUC) of 0.93. After 1st/99th-percentile truncation, the maximum stabilized weight was 10.67 and the effective sample size within the complete-case sample was approximately 7,107, or 36.0% of nominal complete-case N. Fatigue and readiness showed similar effective-sample-size loss. The integrity audit also showed that about 15.8% of complete-case rows used a recent wellness value from t-2 or t-3 while the lag-day reporting indicator used by the weighting procedure referred to t-1. These weights therefore do not justify interpreting IPW as a correction that removes selection bias; the weighted results are retained only as sensitivity analyses.

Across the scientifically reported model space, 49 successfully fitted model specifications contributed 147 focal exposure/interaction tests, including 30 load x wellness interaction tests; 48 focal tests and 5 interaction tests had nominal p < 0.05. These tests are not independent because the specifications overlap and reuse the same sparse event set. Internal quality-assurance fits, failed fits, and rejected fallback levels were excluded from these reporting denominators and remain fully documented in the reproducibility archive (S2 File). Accordingly, isolated nominal p values are interpreted only in conjunction with effect stability, precision, and the fixed information limit of 28 complete-case onset-days from nine event players.

## Discussion

### Principal findings

This reanalysis does not provide stable evidence that within-player deviations in recent training load or registered load x wellness interaction hypotheses are associated with next-day onset of self-reported lower-limb pain episodes. Retrospective full-period scaling produced an inverse acute-load x soreness estimate in Firth and the IPW sensitivity model, but cluster bootstrap intervals were wider, the event set was very small, the weighting procedure retained a residual lag-alignment limitation, and the interaction attenuated to approximately the null when within-player standardization was recalculated using only information available before each day. The same backward-looking sensitivity also changed the chronic-load estimate from near null to above 1.0. That degree of specification sensitivity is more informative than whether any single nominal interval excludes 1.0.

Previous episode history had the largest estimated main-effect association in several models, which is biologically plausible and consistent with broader injury literature [7,24]. Nevertheless, the estimate was imprecise and partly sensitive to model structure, so it should be regarded as a marker of vulnerability and data structure rather than a validated prediction rule. Importantly, multiple recurrent-event estimators and sensitivity models all reuse the same limited outcome information; fitting more models cannot compensate for 28 complete-case onset-days from nine event players.

### Biological and practical interpretation

The registered interaction hypothesis was biologically plausible: a higher-than-usual recent load combined with worse-than-usual soreness could represent incomplete recovery, transient tissue sensitivity, or a period in which the same external task produces greater internal strain. Training-load studies in women players show that daily load can relate to next-day fatigue and soreness, but evidence connecting these fluctuations to injury remains sparse [3]. More broadly, football training design can alter internal load, performance, and enjoyment even when the nominal training objective is similar [10,11], and movement/performance characteristics in female players are multidimensional rather than reducible to one screening score [9]. These observations support monitoring as contextual information, but not as justification for a universal injury-risk threshold.

The inverse soreness interaction under the retrospective scaling has no convincing protective interpretation. A value below 1.0 could arise from training modification after soreness, participation patterns, reporting behavior, mathematical conditioning on who remains available to train, or the construction of the within-player reference distribution. The disappearance of the interaction under backward-looking standardization makes a protective biological explanation especially difficult to defend. For practice, the result argues against using this dataset to tell staff that high acute load is safer on high-soreness days. It also argues against the opposite claim that a specific load-wellness combination reliably identifies imminent pain onset.

The backward-looking chronic-load result deserves the same restraint. Its change of direction indicates that the meaning of a one-standard-deviation load contrast depends strongly on whether the reference distribution uses the player’s entire observed season or only past information. The sensitivity was added because a real-time practitioner could not know a player’s future mean and standard deviation. It should not be read as discovering a new causal chronic-load effect; rather, it demonstrates that the original retrospective coefficient was not invariant to a clinically relevant reparameterization.

### Relation to previous literature

Our findings fit a literature in which athlete-monitoring associations are usually small, heterogeneous, and sensitive to how load, wellness, and injury are defined [1–4]. In elite women’s football specifically, systematic reviews emphasize both the burden of lower-limb injury and the limited evidence linking routinely monitored training load to subsequent injury [3,5,6]. Prospective risk-factor syntheses in female athletes identify previous injury as a recurring marker but do not support a simple one-variable screening paradigm [7]. Injury-prevention trials show that structured exercise programmes can reduce injury incidence [8], which is a different evidentiary claim from predicting tomorrow’s symptom onset from a monitoring dashboard.

Recent studies broaden this context but should not be overextended. Alexe et al. examined functional movement quality and performance in elite female youth players [9]; Rahmoune et al. compared small-sided games with a dribbling circuit in youth players [10]; and Doua et al. compared reactive and planned agility training with attention to physical performance, internal load, and enjoyment [11]. Together they illustrate how performance capacity and internal response vary with athlete and training context. None provides evidence that a particular daily load-wellness threshold predicts lower-limb injury, which is precisely why longitudinal event-based analyses such as the present study remain needed.

### Methodological implications

Three methodological points are relevant to interpretation. Wellness polarity should be verified from the instrument rather than inferred from associations; here, soreness direction was confirmed from independent PmSys documentation without using outcome information. Recurrent symptom reports require an explicit risk-set definition, and retrospective within-player standardization should not be conflated with a value that would have been available prospectively on the same day. These points support transparent use of athlete-monitoring data without implying that methodological complexity can overcome sparse outcome information.

### Strengths and limitations

Strengths include an unchanged public preregistration; explicit separation of preregistered, frozen revision-stage, and later sensitivity analyses; raw-data re-derivation with numerical reconciliation; outcome-blind polarity verification; an explicit recurrent-event risk set; and targeted sparse-data, separation, and exposure-construction diagnostics. Complete code and reproducibility materials are provided in S2 File.

The principal limitation is information size. Only 42 onset-days from 15 players entered the risk set, and only 28 onset-days from nine event players entered the complete-case interaction models. With approximately eight to nine fitted coefficients in several focal models, crude events-per-parameter values were around 3–4. We therefore do not treat asymptotic p values as confirmatory, and Firth penalization cannot create information that is absent from the data. Whole-player bootstrap resampling reflects within-player dependence but remains fragile with only nine complete-case event players.

Second, event information was concentrated by player, team, and season. Team B had no complete-case onset event, producing complete separation; 71% of complete-case events occurred in 2020, and season terms were wide or aliased in some recurrent-event estimators. Targeted team- and season-removal analyses did not explain the full-period soreness interaction, but they demonstrate limited support for estimating team or season effects.

Third, monitoring was incomplete. Approximately 54% of risk-set days entered the complete-case wellness models, and IPW effective sample sizes were only about 36% of nominal complete-case N. The weighting procedure also retained a partial lag-alignment limitation: in approximately 15.8% of complete-case rows, the recent-3-day wellness value originated from t-2 or t-3 while the reporting indicator used by the weight referred to t-1. Selection can therefore depend on unmeasured or unobserved wellness itself, and the weighted models cannot establish that missing-data bias was removed.

Fourth, the outcome and wellness exposures come from the same self-report system. The episode outcome is a subjective symptom report rather than a clinician-confirmed diagnosis [12], and reporting may depend on symptoms, availability, team routines, and perceived wellness. Fifth, acute and chronic windows overlap mathematically in the reference exposure definition. Although VIFs were not extreme and a non-overlapping chronic window produced similar focal estimates, the coefficients should not be interpreted as fully independent physiological components.

Sixth, retrospective full-period within-player standardization uses information that would not be available in real time. The backward-looking sensitivity materially changed the focal interaction and chronic-load estimates, so real-time implementation cannot be inferred from the retrospective full-period coefficients. Finally, the public dataset contains no age, anthropometric, position, or clinical diagnostic metadata, and it covers only two elite Norwegian women’s teams across two seasons, including the COVID-19-disrupted 2020 season. Generalization to other teams, leagues, age groups, or injury definitions is therefore limited.

## Conclusions

In this reproducible secondary analysis of SoccerMon, the available data did not provide stable evidence that within-player deviations in recent training load or registered load x wellness interaction hypotheses were associated with next-day onset of self-reported lower-limb pain/injury episodes. An inverse acute-load x soreness estimate appeared under retrospective full-period standardization, but it was imprecise under player-cluster resampling and attenuated to approximately the null under backward-looking standardization. The same sensitivity materially changed the chronic-load coefficient. Previous episode history showed the largest estimated association, but uncertainty remained substantial. Given 28 complete-case onset-days from nine event players, concentration of episodes and onset-days in a small number of players, missing monitoring, complete team separation, seasonal imbalance, and a broad overlapping analysis space, these results should constrain rather than amplify claims. Practically, load and wellness signals are better interpreted longitudinally and in context than converted into isolated next-day symptom-risk cut-points. Future studies should evaluate prospectively defined, backward-looking monitoring baselines in larger multi-team cohorts with more outcome events and standardized symptom or clinical outcome ascertainment.

## Supporting information

S1 Text STROBE checklist. (DOCX)

S1 Fig Interaction estimates across fitted estimators. (TIF)

S1 Table Training-load and session descriptives. (DOCX)

S2 Table Wellness descriptives and canonical coding. (DOCX)

S3 Table Exposure correlations and collinearity diagnostics. (DOCX)

S4 Table Model diagnostics. (DOCX)

S5 Table Full model coefficients. (DOCX)

S6 Table Washout sensitivity analyses. (DOCX)

S7 Table Additional sensitivity analyses. (DOCX)

S8 Table Bootstrap and cluster-robust inference. (DOCX)

S9 Table Missing-data and IPW analyses. (DOCX)

S10 Table Protocol and analysis-plan reconciliation. (DOCX)

S1 File Code reproduction record. (DOCX)

S2 File Reproducibility archive. (ZIP)

S3 File Original OSF preregistration protocol. (PDF)
