# Supplementary material for: Within-player load-wellness monitoring and lower-limb pain in elite female football: A reproducible secondary analysis of the SoccerMon dataset
Source: PLoS One. 2026 Nov 13;21(11):e0362663. doi: 10.1371/journal.pone.0362663 (PMC13766280; doi:10.1371/journal.pone.0362663)
Supplement: Supplementary file 1 [file pone.0362663.s015.pdf]

Do Within-Player Deviations in Training Load and Wellness Precede Self-Reported Lower-Limb Injury or Pain Episodes in Elite Female Football Players?

A Secondary Longitudinal Analysis of the SoccerMon Dataset

Protocol version: Final OSF preregistration version | Date: 29 May 2026

**Registration platform**	OSF. Preferred template: Secondary Data Preregistration. If unavailable, use OSF Preregistration or Open-Ended Registration.
**Study design**	Retrospective secondary longitudinal analysis of prospectively collected athlete-monitoring data.
**Dataset**	SoccerMon dataset, open-access Zenodo record DOI: 10.5281/zenodo.10033832; data descriptor DOI: 10.1038/s41597-024-03386-x.
**Data files planned for the primary analysis**	Subjective training-load, wellness, and self-reported injury files. GPS-derived objective reports, illness reports, and game-performance files will not be used in the primary analysis.
**Primary outcome**	New onset of a self-reported lower-limb injury or pain episode.
**Exploratory outcome**	New onset of a self-reported knee-region injury or pain episode.
**Main exposure concept**	Within-player deviations in training load and wellness, using lagged values and rolling summaries preceding the outcome day.

1. Background and rationale

Athlete-monitoring systems are widely used to collect repeated information on training load, wellness, and health status. However, many analyses of sports injury risk focus on between-athlete comparisons or black-box prediction models, which may be less informative for day-to-day applied practice. This study will use the SoccerMon dataset to examine whether short-term within-player deviations in training load and wellness precede the onset of self-reported lower-limb injury or pain episodes in elite female football players.

The focus is explanatory rather than predictive. The central practical question is whether a player is at higher subsequent risk when her recent load or wellness is worse than her own usual level. Lower-limb episodes will be used as the primary outcome because they are more suitable for stable analysis than knee-only episodes. Knee-region episodes will be evaluated only as exploratory analyses.

2. Objectives and hypotheses

2.1 Primary research question

Among elite female football players in the SoccerMon subjective monitoring dataset, do within-player deviations in recent training load and wellness precede the onset of self-reported lower-limb injury or pain episodes?

2.2 Secondary/exploratory research questions

1. Are worse-than-usual soreness, fatigue, or readiness associated with subsequent lower-limb episode onset after accounting for recent training load?
2. Does worse-than-usual wellness modify the association between higher-than-usual acute training load and subsequent lower-limb episode onset?
3. Are similar patterns observed for knee-region self-reported injury or pain episodes? This analysis will be considered exploratory because of expected sparse events and concentration of knee reports within a limited number of players.

2.3 Hypotheses

1. Higher-than-usual acute training load will be associated with a higher probability of subsequent lower-limb self-reported injury or pain episode onset.
2. Worse-than-usual wellness, particularly higher soreness, higher fatigue, and lower readiness, will be associated with a higher probability of subsequent lower-limb episode onset.
3. The association between higher-than-usual acute training load and episode onset will be stronger when wellness is worse than usual, particularly when soreness is higher than usual.

3. Data source

The study will use the publicly available SoccerMon dataset, which contains anonymized athlete-monitoring data from two elite women's football teams in Norway across the 2020 and 2021 seasons. The preregistered primary analysis will use the subjective monitoring component: training-load reports, wellness reports, and self-reported injury records. The data source will be documented in the final manuscript using the Zenodo record DOI, the data descriptor DOI, the dataset version where available, and the date of access/download.

A preliminary feasibility audit was conducted before preregistration to inspect file structure, variable availability, date formats, player identifiers, body-location labels, and approximate event counts. No inferential models, exposure-outcome association tests, hypothesis-driven subgroup analyses, or model-based comparisons have been conducted before this preregistration.

4. Study design and analytic sample

This is a retrospective secondary longitudinal analysis of prospectively collected athlete-monitoring data. No new participants will be recruited, no intervention will be delivered, and no contact with players will occur.

The unit of analysis will be the player-day. The analytic sample will include SoccerMon players with linkable player identifiers and valid dates across the training-load, wellness, and injury files. Each player's observation period will begin on the date of the first valid subjective monitoring record and end on the date of the last valid subjective monitoring record. Player-days with missing or unresolvable player identifiers, missing or invalid dates, or impossible chronological ordering will be excluded.

The primary analysis will be restricted to days at risk for a new lower-limb episode. Days occurring during an ongoing episode will not be treated as new independent events. A new episode will be allowed only after the prespecified episode washout rule described below.

5. Outcome definitions

5.1 Injury/pain report

Injury records in SoccerMon are self-reported athlete notifications of pain or injury location and severity. They will not be interpreted as clinician-confirmed medical diagnoses. The manuscript will avoid terms implying clinical diagnosis unless the dataset itself provides such information.

5.2 Anatomical mapping

The primary anatomical region will be the lower limb. Lower-limb locations will include available SoccerMon body-location labels corresponding to hip/groin, thigh, knee, lower leg, ankle/foot, and related left/right labels. Non-lower-limb locations such as head/neck or trunk/back will not contribute to the primary lower-limb outcome. The exact mapping from raw body-location labels to anatomical regions will be documented in the analysis codebook before model fitting.

5.3 Episode construction

Reports will be converted into injury/pain episodes before analysis. For a given player and anatomical location, reports separated by no more than 7 days will be merged into the same episode. The first date in the merged sequence will be the episode onset date. Same-day duplicate records for the same player and same location will be collapsed. If a report contains multiple locations, each location will be processed separately before region-level summaries are created.

The primary outcome will be a binary player-day indicator for new onset of any lower-limb self-reported injury or pain episode. The exploratory knee-region outcome will be a binary player-day indicator for new onset of a knee-region self-reported injury or pain episode. A 7-day episode washout will be used in the primary analysis. Alternative washout periods of 3 and 14 days will be examined in sensitivity analyses.

6. Exposure definitions

6.1 Training-load variables

Session rating of perceived exertion (sRPE) will be calculated as session duration multiplied by the reported RPE when required by the raw data structure. Daily training load will be the sum of sRPE for all recorded sessions on a given player-day. The primary acute training-load variable will be the 7-day rolling mean of daily training load over days t-7 to t-1, excluding the outcome day. The primary chronic training-load variable will be the 28-day rolling mean over days t-28 to t-1, excluding the outcome day.

ACWR will not be used as the sole primary exposure. If calculated, ACWR will be defined as acute training load divided by chronic training load and will be used only in sensitivity analyses.

6.2 Wellness variables

Primary wellness variables will be soreness, fatigue, and readiness. Soreness and fatigue will be coded so that higher values indicate worse status. Readiness will be reverse-coded for the primary models so that higher values indicate worse-than-usual readiness. Sleep duration, sleep quality, stress, and mood may be summarized descriptively or examined in exploratory analyses; they will not be included in the main confirmatory model unless prespecified model simplification requires replacing an unavailable primary wellness variable.

6.3 Lagging and temporal ordering

To avoid same-day ambiguity and future-data leakage, exposure values used to predict episode onset on day t will be lagged. Training-load rolling summaries will end on day t-1. Wellness variables will primarily use the most recent available wellness report from day t-1; if day t-1 is unavailable, a prespecified recent-window rule of up to 3 days may be used and will be reported. Sensitivity analyses may use 3-day rolling mean wellness summaries ending on day t-1.

6.4 Within-player deviation

The main exposure parameterization will use within-player deviations. For each player, each exposure will be centered on that player's own mean and, where appropriate, standardized by that player's own standard deviation. Thus, coefficients will be interpreted as the association of being higher or worse than one's own usual level, rather than differences between players. If a player has insufficient within-player variation for a given exposure, that exposure for that player will be set to missing for models requiring the standardized variable.

7. Covariates and confounding control

The primary model will include a player-level random intercept to account for repeated measures within players. Prespecified covariates will include team, season/calendar week or season phase, and previous injury history or recent lower-limb episode status where estimable. Calendar time will be included to reduce confounding by season phase and monitoring period. Additional covariates will be added only if they are available, interpretable, and do not cause model instability given the event count.

8. Primary statistical analysis

The primary analysis will use a mixed-effects logistic regression model with player-day as the unit of analysis and a player-level random intercept. The outcome will be new lower-limb episode onset on day t. Predictors will include lagged within-player deviations in acute training load, chronic training load, soreness, fatigue, and reverse-coded readiness, with the rolling exposure windows ending before day t.

The primary main-effects model will estimate the associations of acute load, chronic load, soreness, fatigue, and readiness with subsequent lower-limb episode onset. A prespecified interaction model will add the interaction between within-player acute load deviation and within-player soreness deviation. If the event count is insufficient or the interaction model is unstable, the main-effects model will be retained as the primary inferential model and the interaction will be reported as exploratory.

Model estimates will be reported as odds ratios with 95% confidence intervals. Predicted probabilities may be plotted for clinically interpretable combinations of recent load and wellness, such as higher-than-usual load with worse-than-usual soreness versus higher-than-usual load with usual or better-than-usual soreness.

9. Model simplification and convergence rules

If the full primary model fails to converge, shows quasi-complete separation, or is too sparse relative to the number of episode onsets, simplification will follow this order: (1) remove interaction terms; (2) retain acute load, soreness, fatigue, readiness, and calendar time while removing chronic load if collinear with acute load; (3) reduce wellness predictors to soreness plus one additional wellness variable chosen a priori in the order fatigue then readiness; (4) fit a fixed-effect or population-average logistic model with robust standard errors clustered by player. Any use of these fallback steps will be explicitly reported.

10. Sensitivity and exploratory analyses

- Episode definition: repeat the primary model using 3-day and 14-day washout rules.
- Severity: repeat analyses using major-pain reports only if event counts permit.
- Anatomical region: examine knee-region episode onset as an exploratory outcome.
- Training-load metric: evaluate ACWR and alternative chronic windows, including 42-day chronic load if data density permits.
- Wellness metric: replace soreness with fatigue or reverse-coded readiness as the primary wellness interaction term.
- Risk-set definition: assess whether excluding days shortly after any injury/pain episode changes estimates.
- Missingness handling: compare complete-case analysis with a conservative alternative in which unreported training-load days are treated as missing rather than zero when the monitoring context does not support a rest-day interpretation.

Exploratory analyses will be clearly labeled and will not be presented as confirmatory evidence. No machine-learning prediction model will be used as the primary analysis.

11. Missing data

Missing outcome dates or unresolvable injury body-location labels will be excluded from region-specific analyses. No imputation will be performed for outcome onsets. The amount and pattern of missingness in training-load and wellness variables will be reported. The primary analysis will use available player-days with complete data for variables required in each model. Multiple imputation is not planned for the primary analysis because the data are intensive longitudinal monitoring data with non-random reporting patterns.

12. Data management and reproducibility

Raw data files will be preserved unchanged. All transformations from raw reports to injury episodes and player-day analytic datasets will be performed by script. The final workflow will generate at least two derived files: an injury episode file and a player-day analysis file. Code will document anatomical mapping, date parsing, episode construction, rolling exposure windows, within-player standardization, exclusion rules, and model specifications.

13. Ethics and interpretation

This study uses anonymized, publicly available secondary data and does not involve new participant recruitment or intervention. The analysis will respect the limitations of the dataset. Outcomes will be described as self-reported injury or pain episodes rather than clinician-confirmed injuries. The study will not infer specific diagnoses such as patellar tendinopathy, anterior knee pain syndrome, or ligament injury unless such diagnoses are explicitly available in the data.

The study will be interpreted as an explanatory longitudinal secondary analysis of athlete-monitoring data. It will not be described as a newly conducted prospective cohort study and will not be framed as a machine-learning prediction study.

14. Planned reporting

The final manuscript will follow relevant STROBE principles for observational research. Primary, sensitivity, and exploratory analyses will be clearly distinguished. Any deviation from this preregistered protocol will be documented, dated, and justified in the manuscript or supplementary materials.

15. References

1. Midoglu C, Winther AK, Boeker M, et al. A large-scale multivariate soccer athlete health, performance, and position monitoring dataset. Scientific Data. 2024;11:553. doi:10.1038/s41597-024-03386-x.
2. Midoglu C, Winther AK, Boeker M, et al. SoccerMon dataset. Zenodo. doi:10.5281/zenodo.10033832.
3. von Elm E, Altman DG, Egger M, et al. The Strengthening the Reporting of Observational Studies in Epidemiology (STROBE) statement: guidelines for reporting observational studies.
