# Supplementary material for: Within-player load-wellness monitoring and lower-limb pain in elite female football: A reproducible secondary analysis of the SoccerMon dataset
Source: PLoS One. 2026 Nov 13;21(11):e0362663. doi: 10.1371/journal.pone.0362663 (PMC13766280; doi:10.1371/journal.pone.0362663)
Supplement: Supplementary file 2 [file pone.0362663.s001.docx]

# S1 Checklist. STROBE checklist (with RECORD extension items for routinely collected data) - PONE-D-26-31950 R2

Retrospective secondary longitudinal analysis of routinely collected athlete-monitoring data (SoccerMon). Locations refer to the revised (R2) manuscript; supporting items are labelled S1-S10 Tables, S1 Fig, and S1-S3 Files. Section names correspond to the headings used in the revised manuscript.

| Item | Recommendation | Location in revised manuscript |
| --- | --- | --- |
| 1 Title/abstract | (a) Design stated in title/abstract; (b) informative abstract | Title; Abstract |
| 2 Background/rationale | Scientific background and rationale | Introduction, paragraphs 1-5 |
| 3 Objectives | Specific objectives and hypotheses | Introduction, final two paragraphs; registered hypotheses in S3 File; S10 Table |
| 4 Study design | Key design elements | Methods - Study design, registration, and analysis hierarchy |
| 5 Setting | Setting, locations, dates | Methods - Data source, participants, and data collection |
| 6 Participants | Eligibility criteria, sources, follow-up | Methods - Data source, participants, and data collection; Methods - Training load, episodes, and risk set (risk-set definition) |
| 7 Variables | Outcomes, exposures, confounders, effect modifiers | Methods - Training load, episodes, and risk set; Primary and alternative exposure parameterization; Statistical analysis |
| 8 Data sources/measurement | Data sources and assessment methods | Methods - Data source, participants, and data collection; Raw-data integrity and polarity verification; S1 File; S2 File |
| 9 Bias | Efforts to address bias | Methods - Raw-data integrity and polarity verification (outcome-blind polarity check); temporal ordering of lagged exposures; Statistical analysis (inverse-probability-of-observation weighting); Discussion - Strengths and limitations |
| 10 Study size | How study size arrived at | Methods - Data source, participants, and data collection (fixed public dataset; 36,550 theoretical player-days); Results - Data structure, event concentration, and missingness; Table 1 |
| 11 Quantitative variables | Handling of quantitative variables | Methods - Primary and alternative exposure parameterization (within-player z-score deviations); S3 Table |
| 12a Statistical methods | All statistical methods | Methods - Statistical analysis; frozen revision-stage analysis plan in S2 File; S10 Table |
| 12b Confounding control | Confounding control | Methods - Statistical analysis (chronic load, previous-episode history, calendar time, team, season, player random intercept) |
| 12c Subgroups/interactions | Subgroups and interactions | Methods - Statistical analysis (registered acute-load x soreness-burden contrast; fatigue and readiness variants); S5 Table |
| 12d Missing data | Missing data handling | Methods - Statistical analysis (model-specific complete-case analysis and IPW); Results - Missing-data diagnostics and multiplicity; S9 Table |
| 12e Loss to follow-up | Loss to follow-up | Not applicable - fixed retrospective calendar grid; reporting windows and risk-set re-entry are described in Methods - Training load, episodes, and risk set |
| 12f Analytical strategy | Sensitivity analyses | Methods - Statistical analysis; Results - Sensitivity analyses of exposure construction and model stability; Table 3; S6-S8 Tables |
| 13a Participants | Numbers at each stage | Results - Data structure, event concentration, and missingness; Table 1 |
| 13b Non-participation | Reasons for non-participation | Results - Data structure, event concentration, and missingness; Results - Missing-data diagnostics and multiplicity; Table 1; S9 Table |
| 13c Flow diagram | Flow diagram | Table 1 (structured sample and event flow); Fig 1 |
| 14a Descriptive data | Characteristics of participants/data | Table 1; S1 Table; S2 Table |
| 14b Missingness | Missing data per variable | Table 1; S2 Table; S9 Table |
| 14c Follow-up time | Follow-up time | Methods - Data source, participants, and data collection (731 days per player); Table 1 |
| 15 Outcome data | Numbers of outcome events | Results - Data structure, event concentration, and missingness; Table 1; Fig 1; Fig 2 |
| 16a Main results | Unadjusted/adjusted estimates with CI | Results - Primary main effects and focal soreness interaction; Table 2; S5 Table |
| 16b Category boundaries | Category boundaries | Not applicable - exposures were modelled as continuous within-player z-score deviations |
| 16c Relative/absolute | Consider translating relative to absolute | Results - Primary main effects and focal soreness interaction; Fig 3 (conditional predicted probabilities, interpreted with explicit caveats) |
| 17 Other analyses | Other analyses | Results - Sensitivity analyses of exposure construction and model stability; Results - Missing-data diagnostics and multiplicity; Table 3; S6-S9 Tables |
| 18 Key results | Key results summarised | Discussion - Principal findings |
| 19 Limitations | Limitations incl. bias direction/magnitude | Discussion - Strengths and limitations |
| 20 Interpretation | Cautious overall interpretation | Discussion - Biological and practical interpretation; Methodological implications; Conclusions |
| 21 Generalisability | Generalisability | Discussion - Strengths and limitations (two teams, two seasons, one national league) |
| 22 Funding | Funding | Acknowledgments (none); no funding was received for this secondary analysis |
| RECORD extension | Additional reporting items for studies using routinely collected health data | Applied where relevant to this secondary analysis |
| RECORD 1.1 | Type of data used and the databases named in the title or abstract | Title; Abstract (routinely collected athlete-monitoring data; SoccerMon) |
| RECORD 1.2 | Geographic region and timeframe stated | Abstract; Methods - Data source, participants, and data collection (Norwegian elite women's league, 2020 and 2021 seasons) |
| RECORD 1.3 | Linkage between databases stated | Not applicable - no database linkage was performed |
| RECORD 6.1 | Methods of study population selection, including codes and algorithms | Methods - Data source, participants, and data collection; Methods - Training load, episodes, and risk set; complete selection and derivation code in S2 File |
| RECORD 6.2 | Validation of the codes or algorithms used for selection | Methods - Raw-data integrity and polarity verification; independent raw-data rebuild, SHA-256 file hashes, and predefined integrity checks reported in S1 File and S2 File |
| RECORD 6.3 | Flow diagram for linkage | Not applicable - no linkage; sample and event flow are reported in Table 1 |
| RECORD 7.1 | Complete list of codes and algorithms used to classify exposures, outcomes, and confounders | Methods - Training load, episodes, and risk set; Methods - Primary and alternative exposure parameterization; full derivation code in S2 File |
| RECORD 12.1 | Extent to which the investigators had access to the database population | Methods - Data source, participants, and data collection; the complete public subjective release was used, with no additional access restriction |
| RECORD 12.2 | Information on data cleaning methods | Methods - Raw-data integrity and polarity verification; S1 File; S2 File |
| RECORD 12.3 | Person-level, institutional-level, or other data linkage | Not applicable - person-level data only; no linkage was performed |
| RECORD 13.1 | Detailed description of the selection of persons included in the study | Methods - Data source, participants, and data collection; Results - Data structure, event concentration, and missingness; Table 1 |
| RECORD 19.1 | Implications of using data that were not created to answer the specific research question | Discussion - Methodological implications; Discussion - Strengths and limitations |
| RECORD 22.1 | Information on how to access the study protocol, raw data, and programming code | Data availability; S1 File; S2 File; S3 File (original OSF preregistration, DOI 10.17605/OSF.IO/UAFWQ) |
