# Supplementary material for: Within-player load-wellness monitoring and lower-limb pain in elite female football: A reproducible secondary analysis of the SoccerMon dataset
Source: PLoS One. 2026 Nov 13;21(11):e0362663. doi: 10.1371/journal.pone.0362663 (PMC13766280; doi:10.1371/journal.pone.0362663)
Supplement: Supplementary file 3 [file pone.0362663.s003.docx]

**S1 Table. Training-load and session descriptives (36,550 player-days).**

| **Variable** | **Mean ± SD** | **Median (IQR)** | **N** |
| --- | --- | --- | --- |
| Daily load (sRPE) | 194.5 ± 298.1 | 0.0 (0.0–360.0) | 36,550 |
| Weekly load | 1349.6 ± 1386.3 | 1130.0 (0.0–2530.0) | 36,550 |
| ATL | 192.8 ± 197.9 | 162.9 (0.0–361.4) | 36,550 |
| CTL28 | 191.1 ± 180.1 | 183.2 (0.0–347.5) | 36,550 |
| CTL42 | 190.3 ± 175.3 | 183.8 (0.0–344.8) | 36,550 |
| ACWR | 0.7 ± 0.8 | 0.7 (0.0–1.1) | 36,550 |
| Monotony | 0.7 ± 0.7 | 0.6 (0.0–1.3) | 36,550 |
| Strain | 1897.4 ± 2465.1 | 806.0 (0.0–3266.0) | 36,550 |
| Sessions per day (reported days) | 1.15 ± 0.38 | 1 | 14,197 |
