# Supplementary material for: Within-player load-wellness monitoring and lower-limb pain in elite female football: A reproducible secondary analysis of the SoccerMon dataset
Source: PLoS One. 2026 Nov 13;21(11):e0362663. doi: 10.1371/journal.pone.0362663 (PMC13766280; doi:10.1371/journal.pone.0362663)
Supplement: Supplementary file 4 [file pone.0362663.s004.docx]

**S2 Table. Wellness descriptives and canonical coding.**

| **Variable** | **Mean ± SD** | **Median** | **N reported (% missing)** | **Canonical coding** |
| --- | --- | --- | --- | --- |
| fatigue | 3.03 ± 0.62 | 3.0 | 16,992 (53.5%) | fatigue_burden = 6 − fatigue |
| readiness | 6.80 ± 1.46 | 7.0 | 16,997 (53.5%) | readiness_deficit = 11 − readiness |
| soreness | 2.80 ± 0.73 | 3.0 | 16,999 (53.5%) | soreness_burden = 6 − soreness |
| mood | 3.37 ± 0.61 | 3.0 | 16,999 (53.5%) | recorded scale (descriptive only) |
| stress | 3.20 ± 0.63 | 3.0 | 16,997 (53.5%) | recorded scale (descriptive only) |
| sleep_quality | 3.26 ± 0.72 | 3.0 | 16,994 (53.5%) | recorded scale (descriptive only) |
| sleep_duration | 8.14 ± 0.99 | 8.0 | 16,981 (53.5%) | recorded scale (descriptive only) |

Note. All PmSys 1-5 items are positively scored (higher raw = better state); readiness is scored 1-10 with higher values indicating greater readiness. Within-player z-scores used in modeling are computed on burden/deficit variables so that higher transformed values consistently indicate a worse-than-usual state.
