# Supplementary material for: Within-player load-wellness monitoring and lower-limb pain in elite female football: A reproducible secondary analysis of the SoccerMon dataset
Source: PLoS One. 2026 Nov 13;21(11):e0362663. doi: 10.1371/journal.pone.0362663 (PMC13766280; doi:10.1371/journal.pone.0362663)
Supplement: Supplementary file 5 [file pone.0362663.s005.docx]

**S3 Table. Exposure correlations and collinearity diagnostics.**

**A. Acute/chronic overlap in complete-case samples**

| **Metric** | **Soreness CC** | **Fatigue CC** | **Readiness CC** | **Interpretation** |
| --- | --- | --- | --- | --- |
| Complete-case N | 19,758 | 19,815 | 19,817 | 28 onset-days in each model |
| Pearson r: acute t-7:t-1 vs chronic t-28:t-1 | 0.6981 (0.6909-0.7052) | 0.6912 (0.6839-0.6984) | 0.6909 (0.6836-0.6981) | Moderate structural overlap |
| Spearman rho: same pair | 0.6751 | 0.6727 | 0.6724 | Rank-based result similar |
| Two-predictor VIF | 1.9506 | 1.9150 | 1.9134 | Below levels usually regarded as severe |

**B. Full-design diagnostics in the soreness complete-case sample**

| **Diagnostic** | **Value** | **Detail** |
| --- | --- | --- |
| VIF: acute load | 2.0136 | Main-effects design |
| VIF: chronic load | 1.9585 | Main-effects design |
| Maximum VIF | 4.0846 | Calendar time; main-effects design |
| Maximum VIF with acute x chronic interaction added | 4.1453 | Calendar time |
| Scaled condition number | 3.7910 | Centered/unit-SD main-effects design |
| Scaled condition number with interaction | 4.3320 | Centered/unit-SD design |
| Within-player demeaned acute/chronic r | 0.6227 | Removes player means |
| Between-player means r | 0.9925 | Descriptive between-player component |

**C. Non-overlapping chronic-window check**

| **Chronic-load definition** | **Pearson r with acute load** | **Spearman rho** | **N / onsets** | **Comment** |
| --- | --- | --- | --- | --- |
| Reference chronic: t-28:t-1 | 0.6981 | 0.6751 | 19,758 / 28 | Seven acute days are nested in the 28-day window |
| Non-overlapping chronic: t-28:t-8 | 0.4695 | 0.4505 | 19,758 / 28 | Acute t-7:t-1 is mathematically disjoint |
| Acute vs raw-lag non-overlap remainder | 0.5410 | 0.5272 | 19,758 / 28 | Derived check before within-player standardization |

**D. Additional Spearman correlations among within-player z exposures**

| **Variable 1** | **Variable 2** | **Spearman rho** |
| --- | --- | --- |
| acute_load_7d_wp_z | chronic_load_28d_wp_z | 0.675 |
| fatigue_burden_recent3_wp_z | readiness_deficit_recent3_wp_z | 0.412 |
| soreness_burden_recent3_wp_z | readiness_deficit_recent3_wp_z | 0.306 |
| fatigue_burden_recent3_wp_z | soreness_burden_recent3_wp_z | 0.296 |
| acute_load_7d_wp_z | soreness_burden_recent3_wp_z | 0.064 |
| chronic_load_28d_wp_z | readiness_deficit_recent3_wp_z | -0.056 |
| acute_load_7d_wp_z | readiness_deficit_recent3_wp_z | -0.051 |
| acute_load_7d_wp_z | fatigue_burden_recent3_wp_z | 0.020 |
| chronic_load_28d_wp_z | soreness_burden_recent3_wp_z | 0.013 |
| chronic_load_28d_wp_z | fatigue_burden_recent3_wp_z | -0.008 |

Note. Correlations and VIFs are descriptive diagnostics, not additional inferential tests. The non-overlapping t-28:t-8 sensitivity is reported in S7 Table. The full main-effects VIFs remained at or below 4.08 and the scaled condition number was 3.79; these values indicate appreciable overlap but not extreme numerical collinearity.
