# Supplementary material for: Within-player load-wellness monitoring and lower-limb pain in elite female football: A reproducible secondary analysis of the SoccerMon dataset
Source: PLoS One. 2026 Nov 13;21(11):e0362663. doi: 10.1371/journal.pone.0362663 (PMC13766280; doi:10.1371/journal.pone.0362663)
Supplement: Supplementary file 6 [file pone.0362663.s006.docx]

**S4 Table. Model diagnostics.**

**A. Reference and small-sample models**

| **Model** | **Wellness** | **Final estimator/status** | **N** | **Players** | **Onsets** | **Fitted parameters** | **Crude EPV** | **Diagnostic note** |
| --- | --- | --- | --- | --- | --- | --- | --- | --- |
| A | Soreness | Population-average logit + player CR2 fallback | 19,758 | 49 | 28 | 8 | 3.50 | All GLMM ladder levels were rejected because of Team B separation; team coefficient not interpretable. |
| A | Fatigue | Population-average logit + player CR2 fallback | 19,815 | 50 | 28 | 8 | 3.50 | All GLMM ladder levels were rejected because of Team B separation; team coefficient not interpretable. |
| A | Readiness | Population-average logit + player CR2 fallback | 19,817 | 50 | 28 | 8 | 3.50 | All GLMM ladder levels were rejected because of Team B separation; team coefficient not interpretable. |
| F | Soreness | Firth bias-reduced logistic | 19,758 | 49 | 28 | 9 | 3.11 | Penalized estimator retained under sparse events; profile-likelihood CI; player-cluster bootstrap in S8. |
| F | Fatigue | Firth bias-reduced logistic | 19,815 | 50 | 28 | 9 | 3.11 | Penalized estimator retained under sparse events; profile-likelihood CI; player-cluster bootstrap in S8. |
| F | Readiness | Firth bias-reduced logistic | 19,817 | 50 | 28 | 9 | 3.11 | Penalized estimator retained under sparse events; profile-likelihood CI; player-cluster bootstrap in S8. |
| IPW | Soreness | Weighted marginal logit + player CR2 | 19,758 | 49 | 28 | 9 | 3.11 | ESS_truncated=7107.2; ESS_over_n=0.36; max_weight=10.665 |
| IPW | Fatigue | Weighted marginal logit + player CR2 | 19,815 | 50 | 28 | 9 | 3.11 | ESS_truncated=7079; ESS_over_n=0.357; max_weight=10.672 |
| IPW | Readiness | Weighted marginal logit + player CR2 | 19,817 | 50 | 28 | 9 | 3.11 | ESS_truncated=7059.2; ESS_over_n=0.356; max_weight=10.625 |

**B. Revision-stage sensitivity models**

| **Analysis** | **Final estimator/status** | **N** | **Players** | **Onsets** | **Fitted parameters** | **Crude EPV** | **Separation / diagnostic** |
| --- | --- | --- | --- | --- | --- | --- | --- |
| 3-day washout | Full mixed-effects logistic (GLMM) | 19,859 | 49 | 48 | 10 | 4.80 | No separation; full GLMM accepted |
| 14-day washout | Population-average logit + player CR2 fallback | 19,664 | 49 | 20 | 8 | 2.50 | Team B separation; population-average fallback |
| Risk-set recovery: end + washout | Population-average logit + player CR2 fallback | 19,517 | 49 | 25 | 8 | 3.12 | Team B separation; population-average fallback |
| ACWR replacement | Population-average logit + player CR2 fallback | 19,635 | 49 | 28 | 8 | 3.50 | Team B separation; population-average fallback |
| Strict t-1 wellness | Population-average logit + player CR2 fallback | 16,646 | 49 | 23 | 8 | 2.88 | Team B separation; population-average fallback |
| Exclude highest episode contributor | Population-average logit + player CR2 fallback | 19,297 | 48 | 22 | 8 | 2.75 | Team B separation; population-average fallback |

**C. Recurrent-event candidate models**

| **Model** | **Wellness** | **Status** | **N** | **Onsets** | **Fitted parameters** | **Crude EPV** | **Aliasing / convergence note** |
| --- | --- | --- | --- | --- | --- | --- | --- |
| Andersen-Gill | Soreness | Converged with warning | 19,758 | 28 | 6 | 4.67 | Aliased/dropped: calendar_time \|\| season2021; warnings: Loglik converged before variable 6 ; beta may be infinite. |
| Andersen-Gill | Fatigue | Converged with warning | 19,815 | 28 | 6 | 4.67 | Aliased/dropped: calendar_time \|\| season2021; warnings: Loglik converged before variable 6 ; beta may be infinite. |
| Andersen-Gill | Readiness | Converged with warning | 19,817 | 28 | 6 | 4.67 | Aliased/dropped: calendar_time \|\| season2021; warnings: Loglik converged before variable 6 ; beta may be infinite. |
| PWP total-time | Soreness | Converged with warning | 19,758 | 28 | 5 | 5.60 | Aliased/dropped: prev_episode_ever \|\| calendar_time \|\| season2021; warnings: Loglik converged before variable 6 ; beta may be infinite. |
| PWP total-time | Fatigue | Converged with warning | 19,815 | 28 | 5 | 5.60 | Aliased/dropped: prev_episode_ever \|\| calendar_time \|\| season2021; warnings: Loglik converged before variable 6 ; beta may be infinite. |
| PWP total-time | Readiness | Converged with warning | 19,817 | 28 | 5 | 5.60 | Aliased/dropped: prev_episode_ever \|\| calendar_time \|\| season2021; warnings: Loglik converged before variable 6 ; beta may be infinite. |
| PWP gap-time | Soreness | Converged with warning | 19,758 | 28 | 7 | 4.00 | Aliased/dropped: prev_episode_ever; warnings: Loglik converged before variable 6 ; beta may be infinite. |
| PWP gap-time | Fatigue | Converged with warning | 19,815 | 28 | 7 | 4.00 | Aliased/dropped: prev_episode_ever; warnings: Loglik converged before variable 6 ; beta may be infinite. |
| PWP gap-time | Readiness | Converged with warning | 19,817 | 28 | 7 | 4.00 | Aliased/dropped: prev_episode_ever; warnings: Loglik converged before variable 6 ; beta may be infinite. |
| Shared-frailty Cox | Soreness | Completed with warnings | 19,758 | 28 | 7 | 4.00 | Aliased/dropped: calendar_time \|\| frailty(player_id); warnings: Inner loop failed to coverge for iterations 1 2 3 4 5 6 |
| Shared-frailty Cox | Fatigue | Completed with warnings | 19,815 | 28 | 7 | 4.00 | Aliased/dropped: season2021 \|\| frailty(player_id); warnings: Inner loop failed to coverge for iterations 1 2 3 4 5 6 |
| Shared-frailty Cox | Readiness | FAILED | 19,817 | 28 | - | - | No starting estimate was successful; frailty and coxme fallback failed. |

**D. Post-SAP targeted sensitivity analyses**

| **Analysis** | **Timing / role** | **N / onsets** | **Status** | **Key diagnostic/result** |
| --- | --- | --- | --- | --- |
| Non-overlapping chronic window | Post-SAP sensitivity | 19,758 / 28 | Firth + drop-team checks completed | Acute/chronic Pearson r fell from 0.698 to 0.470; Firth interaction OR 0.630 (0.445-0.966). |
| Drop team | Post-SAP sensitivity | 19,758 / 28 | Full GLMM became estimable | Separation resolved; GLMM interaction OR 0.694 (0.470-1.025); Firth 0.671 (0.474-1.069). |
| Drop season | Post-SAP sensitivity | 19,758 / 28 | Completed | Firth interaction OR 0.630 (0.444-0.970); season imbalance remained a precision limitation. |
| Backward-looking expanding-history z-scores | Post-SAP sensitivity | 19,216 / 27 | Completed | No future observations used; Firth interaction OR 0.974 (0.728-1.252); chronic OR 1.955 (1.208-3.058). |
| IPW observation model without onset | Post-SAP sensitivity | 19,758 / 28 (soreness) | Completed | Soreness interaction OR 0.657 (0.425-1.015); ESS 7,115; max truncated weight 10.621. |

Note. EPV is reported only as a descriptive sparsity metric; no 10-EPV rule was used for variable selection. Multiple estimators reuse the same limited event information.
