# Supplementary material for: Within-player load-wellness monitoring and lower-limb pain in elite female football: A reproducible secondary analysis of the SoccerMon dataset
Source: PLoS One. 2026 Nov 13;21(11):e0362663. doi: 10.1371/journal.pone.0362663 (PMC13766280; doi:10.1371/journal.pone.0362663)
Supplement: Supplementary file 7 [file pone.0362663.s007.docx]

**S5 Table. Full coefficients for prioritized Firth and Andersen-Gill wellness models.**

Population-average reference main-effect estimates are prioritized in main-text Table 2; IPW estimates and diagnostics are reported in S9 Table.

**A. Soreness model**

| **Estimator** | **Term** | **Effect ratio** | **95% CI** | **p** | **Interpretation note** |
| --- | --- | --- | --- | --- | --- |
| Firth logistic (OR) | Acute load (7-day within-player z-score) | 0.599 | 0.325–1.114 | 0.105 | — |
| Firth logistic (OR) | Soreness burden (recent 3-day within-player z-score) | 0.823 | 0.579–1.166 | 0.273 | — |
| Firth logistic (OR) | Chronic load (28-day within-player z-score) | 0.947 | 0.533–1.719 | 0.856 | — |
| Firth logistic (OR) | Previous episode history | 6.129 | 2.580–17.424 | <0.001 | — |
| Firth logistic (OR) | Calendar time (per day) | 0.992 | 0.987–0.997 | <0.001 | — |
| Firth logistic (OR) | Team B vs Team A | 0.023 | 0.000–0.170 | <0.001 | Complete separation; bias-reduced estimate—interpret cautiously. |
| Firth logistic (OR) | 2021 season vs 2020 | 2.889 | 0.599–14.053 | 0.185 | — |
| Firth logistic (OR) | Acute load × soreness burden | 0.630 | 0.445–0.965 | 0.035 | — |
| Andersen–Gill Cox (HR) | Acute load (7-day within-player z-score) | 0.478 | 0.225–1.015 | 0.055 | — |
| Andersen–Gill Cox (HR) | Soreness burden (recent 3-day within-player z-score) | 0.713 | 0.426–1.193 | 0.197 | — |
| Andersen–Gill Cox (HR) | Chronic load (28-day within-player z-score) | 0.949 | 0.342–2.636 | 0.920 | — |
| Andersen–Gill Cox (HR) | Previous episode history | 6.374 | 1.724–23.560 | 0.005 | — |
| Andersen–Gill Cox (HR) | Calendar time (per day) | Not estimable | Not estimable | Not estimable | Aliased and dropped from the fitted model. |
| Andersen–Gill Cox (HR) | Team B vs Team A | Not interpretable | Not interpretable | Not interpretable | Complete separation: no Team B onset in the complete-case sample. |
| Andersen–Gill Cox (HR) | 2021 season vs 2020 | Not estimable | Not estimable | Not estimable | Aliased and dropped from the fitted model. |
| Andersen–Gill Cox (HR) | Acute load × soreness burden | 0.589 | 0.402–0.865 | 0.007 | — |

**B. Fatigue model**

| **Estimator** | **Term** | **Effect ratio** | **95% CI** | **p** | **Interpretation note** |
| --- | --- | --- | --- | --- | --- |
| Firth logistic (OR) | Acute load (7-day within-player z-score) | 0.615 | 0.329–1.159 | 0.133 | — |
| Firth logistic (OR) | Fatigue burden (recent 3-day within-player z-score) | 0.759 | 0.529–1.086 | 0.133 | — |
| Firth logistic (OR) | Chronic load (28-day within-player z-score) | 0.949 | 0.532–1.728 | 0.862 | — |
| Firth logistic (OR) | Previous episode history | 5.923 | 2.500–16.804 | <0.001 | — |
| Firth logistic (OR) | Calendar time (per day) | 0.992 | 0.987–0.997 | <0.001 | — |
| Firth logistic (OR) | Team B vs Team A | 0.026 | 0.000–0.190 | <0.001 | Complete separation; bias-reduced estimate—interpret cautiously. |
| Firth logistic (OR) | 2021 season vs 2020 | 2.790 | 0.589–13.267 | 0.194 | — |
| Firth logistic (OR) | Acute load × fatigue burden | 0.819 | 0.569–1.380 | 0.447 | — |
| Andersen–Gill Cox (HR) | Acute load (7-day within-player z-score) | 0.552 | 0.239–1.277 | 0.165 | — |
| Andersen–Gill Cox (HR) | Fatigue burden (recent 3-day within-player z-score) | 0.771 | 0.588–1.011 | 0.060 | — |
| Andersen–Gill Cox (HR) | Chronic load (28-day within-player z-score) | 0.908 | 0.333–2.476 | 0.850 | — |
| Andersen–Gill Cox (HR) | Previous episode history | 6.328 | 1.781–22.482 | 0.004 | — |
| Andersen–Gill Cox (HR) | Calendar time (per day) | Not estimable | Not estimable | Not estimable | Aliased and dropped from the fitted model. |
| Andersen–Gill Cox (HR) | Team B vs Team A | Not interpretable | Not interpretable | Not interpretable | Complete separation: no Team B onset in the complete-case sample. |
| Andersen–Gill Cox (HR) | 2021 season vs 2020 | Not estimable | Not estimable | Not estimable | Aliased and dropped from the fitted model. |
| Andersen–Gill Cox (HR) | Acute load × fatigue burden | 0.784 | 0.595–1.032 | 0.083 | — |

**C. Readiness model**

| **Estimator** | **Term** | **Effect ratio** | **95% CI** | **p** | **Interpretation note** |
| --- | --- | --- | --- | --- | --- |
| Firth logistic (OR) | Acute load (7-day within-player z-score) | 0.682 | 0.364–1.277 | 0.232 | — |
| Firth logistic (OR) | Readiness deficit (recent 3-day within-player z-score) | 1.019 | 0.719–1.420 | 0.915 | — |
| Firth logistic (OR) | Chronic load (28-day within-player z-score) | 0.982 | 0.545–1.799 | 0.951 | — |
| Firth logistic (OR) | Previous episode history | 5.850 | 2.461–16.634 | <0.001 | — |
| Firth logistic (OR) | Calendar time (per day) | 0.992 | 0.987–0.997 | <0.001 | — |
| Firth logistic (OR) | Team B vs Team A | 0.027 | 0.000–0.196 | <0.001 | Complete separation; bias-reduced estimate—interpret cautiously. |
| Firth logistic (OR) | 2021 season vs 2020 | 2.554 | 0.540–12.116 | 0.235 | — |
| Firth logistic (OR) | Acute load × readiness deficit | 0.866 | 0.636–1.270 | 0.427 | — |
| Andersen–Gill Cox (HR) | Acute load (7-day within-player z-score) | 0.554 | 0.242–1.267 | 0.162 | — |
| Andersen–Gill Cox (HR) | Readiness deficit (recent 3-day within-player z-score) | 0.778 | 0.592–1.023 | 0.073 | — |
| Andersen–Gill Cox (HR) | Chronic load (28-day within-player z-score) | 0.922 | 0.328–2.591 | 0.878 | — |
| Andersen–Gill Cox (HR) | Previous episode history | 6.110 | 1.701–21.953 | 0.006 | — |
| Andersen–Gill Cox (HR) | Calendar time (per day) | Not estimable | Not estimable | Not estimable | Aliased and dropped from the fitted model. |
| Andersen–Gill Cox (HR) | Team B vs Team A | Not interpretable | Not interpretable | Not interpretable | Complete separation: no Team B onset in the complete-case sample. |
| Andersen–Gill Cox (HR) | 2021 season vs 2020 | Not estimable | Not estimable | Not estimable | Aliased and dropped from the fitted model. |
| Andersen–Gill Cox (HR) | Acute load × readiness deficit | 0.837 | 0.622–1.127 | 0.241 | — |

Note. Effect ratios are odds ratios (ORs) for Firth logistic rows and hazard ratios (HRs) for Andersen-Gill Cox rows; these model-specific measures are not treated as interchangeable estimands. Firth rows show profile-likelihood 95% CIs; player-cluster bootstrap intervals are reported separately in S8 Table. Andersen-Gill rows use player-cluster-robust standard errors. Calendar time and season were aliased in the Andersen-Gill fits and therefore were not estimated. The Team B coefficient is not substantively interpretable because no Team B onset occurred in the complete-case sample.
