# Supplementary material for: Within-player load-wellness monitoring and lower-limb pain in elite female football: A reproducible secondary analysis of the SoccerMon dataset
Source: PLoS One. 2026 Nov 13;21(11):e0362663. doi: 10.1371/journal.pone.0362663 (PMC13766280; doi:10.1371/journal.pone.0362663)
Supplement: Supplementary file 8 [file pone.0362663.s008.docx]

**S6 Table. Washout sensitivity analyses.**

**A. Three-day washout sensitivity**

Soreness model; complete-case at-risk days = 19,859; onset-days = 48. The full GLMM interaction model was accepted without separation.

| **Term** | **OR** | **95% CI** | **p** | **Term type** |
| --- | --- | --- | --- | --- |
| Acute load (7-day within-player z-score) | 0.721 | 0.436–1.192 | 0.202 | Main effect |
| Soreness burden (recent 3-day within-player z-score) | 0.685 | 0.530–0.887 | 0.004 | Main effect |
| Chronic load (28-day within-player z-score) | 0.863 | 0.519–1.433 | 0.569 | Main effect |
| Previous episode history | 4.291 | 1.101–16.722 | 0.036 | Main effect |
| Calendar time (per day) | 0.988 | 0.983–0.992 | <0.001 | Main effect |
| Team B vs Team A | 0.025 | 0.001–0.446 | 0.012 | Main effect |
| 2021 season vs 2020 | 5.824 | 1.241–27.334 | 0.025 | Main effect |
| Acute load × soreness burden | 0.794 | 0.566–1.112 | 0.179 | Interaction |

Note. OR, odds ratio. The interaction term was retained in this model.

**B. Fourteen-day washout sensitivity**

Soreness model; complete-case at-risk days = 19,664; onset-days = 20. The GLMM ladder remained separated on team and the population-average fallback was used.

| **Term** | **OR** | **95% CI** | **p** | **Term type** |
| --- | --- | --- | --- | --- |
| Acute load (7-day within-player z-score) | 0.895 | 0.270–2.968 | 0.857 | Main effect |
| Soreness burden (recent 3-day within-player z-score) | 0.765 | 0.379–1.543 | 0.453 | Main effect |
| Chronic load (28-day within-player z-score) | 0.838 | 0.430–1.632 | 0.604 | Main effect |
| Previous episode history | 4.388 | 1.140–16.880 | 0.031 | Main effect |
| Calendar time (per day) | 0.994 | 0.989–0.999 | 0.012 | Main effect |
| Team B vs Team A | 0.000 | 0.000–0.000 | <0.001 | Main effect |
| 2021 season vs 2020 | 2.066 | 0.400–10.662 | 0.386 | Main effect |

Note. The interaction term was dropped under the simplification chain in the frozen revision-stage SAP. Boundary estimates displayed as 0.000 are rounded near-zero estimates caused by complete separation and are not interpreted as exact zero.
