# Supplementary material for: Within-player load-wellness monitoring and lower-limb pain in elite female football: A reproducible secondary analysis of the SoccerMon dataset
Source: PLoS One. 2026 Nov 13;21(11):e0362663. doi: 10.1371/journal.pone.0362663 (PMC13766280; doi:10.1371/journal.pone.0362663)
Supplement: Supplementary file 9 [file pone.0362663.s009.docx]

**S7 Table. Additional sensitivity analyses.**

**A. Risk-set re-entry after episode end plus washout**

Soreness model; complete-case at-risk days = 19,517; onset-days = 25.

| **Term** | **OR** | **95% CI** | **p** | **Term type** |
| --- | --- | --- | --- | --- |
| Acute load (7-day within-player z-score) | 0.518 | 0.253–1.062 | 0.072 | Main effect |
| Soreness burden (recent 3-day within-player z-score) | 0.829 | 0.488–1.409 | 0.489 | Main effect |
| Chronic load (28-day within-player z-score) | 0.924 | 0.537–1.590 | 0.776 | Main effect |
| Previous episode history | 6.491 | 1.708–24.666 | 0.006 | Main effect |
| Calendar time (per day) | 0.991 | 0.986–0.997 | 0.004 | Main effect |
| Team B vs Team A | 0.000 | 0.000–0.000 | <0.001 | Main effect |
| 2021 season vs 2020 | 3.031 | 0.354–25.975 | 0.312 | Main effect |

Note. Revision-stage sensitivity. Team separation remained and the population-average fallback was used; the interaction term was dropped under the frozen revision-stage SAP simplification chain.

**B. ACWR-replacement sensitivity**

Soreness model; complete-case at-risk days = 19,635; onset-days = 28.

| **Term** | **OR** | **95% CI** | **p** | **Term type** |
| --- | --- | --- | --- | --- |
| Acute load (7-day within-player z-score) | 0.804 | 0.558–1.159 | 0.242 | Main effect |
| Soreness burden (recent 3-day within-player z-score) | 0.747 | 0.449–1.241 | 0.260 | Main effect |
| Chronic load (28-day within-player z-score) | 0.659 | 0.259–1.673 | 0.380 | Main effect |
| Previous episode history | 6.301 | 1.608–24.695 | 0.008 | Main effect |
| Calendar time (per day) | 0.992 | 0.986–0.997 | 0.002 | Main effect |
| Team B vs Team A | 0.000 | 0.000–0.000 | <0.001 | Main effect |
| 2021 season vs 2020 | 2.779 | 0.389–19.835 | 0.308 | Main effect |

Note. Revision-stage sensitivity. The source model retained the archived term labels for reproducibility; team separation remained and the population-average fallback was used.

**C. Strict t-1 wellness sensitivity**

Soreness model; complete-case at-risk days = 16,646; onset-days = 23.

| **Term** | **OR** | **95% CI** | **p** | **Term type** |
| --- | --- | --- | --- | --- |
| Acute load (7-day within-player z-score) | 0.703 | 0.292–1.697 | 0.434 | Main effect |
| Soreness burden (lag-1 within-player z-score) | 0.723 | 0.442–1.185 | 0.199 | Main effect |
| Chronic load (28-day within-player z-score) | 0.784 | 0.426–1.443 | 0.434 | Main effect |
| Previous episode history | 5.077 | 1.190–21.665 | 0.028 | Main effect |
| Calendar time (per day) | 0.995 | 0.990–0.999 | 0.015 | Main effect |
| Team B vs Team A | 0.000 | 0.000–0.000 | <0.001 | Main effect |
| 2021 season vs 2020 | 1.043 | 0.158–6.873 | 0.965 | Main effect |

Note. Revision-stage sensitivity. Team separation remained and the population-average fallback was used; the interaction term was dropped under the frozen revision-stage SAP simplification chain.

**D. Event concentration and exclusion of the highest episode contributor**

| **Event unit** | **Total** | **Largest single-player contribution** | **Percentage** | **Comment** |
| --- | --- | --- | --- | --- |
| Body-location episodes | 92 | 45 | 48.9% | Episode-concentration metric |
| Risk-set onset-days | 42 | 11 | 26.2% | Player-day unit used for primary inference |
| Soreness complete-case onset-days | 28 | 6 (two players tied) | 21.4% | Complete-case interaction sample |

The pre-existing influence sensitivity below excluded the player with 45 of 92 body-location episodes; this is not the same player as the 11-of-42 risk-set onset-day contributor.

| **Term** | **OR** | **95% CI** | **p** | **Term type** |
| --- | --- | --- | --- | --- |
| Acute load (7-day within-player z-score) | 0.714 | 0.249–2.048 | 0.531 | Main effect |
| Soreness burden (recent 3-day within-player z-score) | 0.741 | 0.361–1.520 | 0.413 | Main effect |
| Chronic load (28-day within-player z-score) | 0.994 | 0.504–1.962 | 0.987 | Main effect |
| Previous episode history | 5.176 | 1.295–20.689 | 0.020 | Main effect |
| Calendar time (per day) | 0.990 | 0.984–0.996 | 0.001 | Main effect |
| Team B vs Team A | 0.000 | 0.000–0.000 | <0.001 | Main effect |
| 2021 season vs 2020 | 6.428 | 1.108–37.293 | 0.038 | Main effect |

Note. This revision-stage influence analysis uses the population-average fallback because team separation persisted. Symmetric leave-one-event-player-out refits were retained as internal QA in S2 File and are not treated as an additional manuscript sensitivity.

**E. Non-overlapping chronic-load window (t-28:t-8)**

| **Estimator** | **Acute load OR (95% CI)** | **Soreness OR (95% CI)** | **Chronic t-28:t-8 OR (95% CI)** | **Previous episode OR (95% CI)** | **Acute x soreness OR (95% CI)** | **N / onsets** |
| --- | --- | --- | --- | --- | --- | --- |
| Firth, team retained | 0.599 (0.358-1.012) | 0.822 (0.579-1.164) | 0.928 (0.593-1.479) | 6.154 (2.590-17.495) | 0.630 (0.445-0.966) | 19,758 / 28 |
| GLMM, team removed | 0.637 (0.388-1.046) | 0.827 (0.574-1.192) | 0.891 (0.541-1.469) | 1.607 (0.330-7.822) | 0.703 (0.475-1.040) | 19,758 / 28 |
| Firth, team removed | 0.653 (0.376-1.134) | 0.852 (0.589-1.227) | 0.781 (0.496-1.263) | 7.106 (3.001-20.153) | 0.670 (0.475-1.069) | 19,758 / 28 |
| Marginal player-cluster bootstrap, team removed | - | - | - | - | 0.740 (0.557-1.133) | 19,758 / 28 |

Note. This post-SAP sensitivity replaced chronic t-28:t-1 with the disjoint t-28:t-8 window, reducing the acute/chronic Pearson correlation from 0.698 to 0.470 without materially changing the retrospective focal interaction.

**F. Team-separation sensitivity: like-for-like Firth comparison**

| **Term** | **Reference Firth with team OR (95% CI)** | **Drop-team Firth OR (95% CI)** | **Interpretation** |
| --- | --- | --- | --- |
| Acute load | 0.599 (0.325-1.114) | 0.705 (0.369-1.347) | Direction retained; intervals remained wide and overlapping. |
| Soreness burden | 0.823 (0.579-1.166) | 0.853 (0.590-1.228) | Direction retained; intervals remained wide and overlapping. |
| Chronic load | 0.947 (0.533-1.719) | 0.749 (0.418-1.385) | Direction retained; intervals remained wide and overlapping. |
| Previous episode history | 6.129 (2.580-17.424) | 7.104 (2.999-20.154) | Largest estimated association in both; wide intervals. |
| Acute x soreness | 0.630 (0.445-0.965) | 0.671 (0.474-1.069) | Direction retained; estimate moved toward null and CI widened. |

Additional drop-team estimates: the full GLMM became estimable after removing team (interaction OR 0.694, 95% CI 0.470-1.025, p=0.066); the marginal player-cluster bootstrap interaction was OR 0.740 (0.556-1.132), P(OR>1)=0.102.

**G. Season instability and drop-season sensitivity**

| **Question / model** | **Key result** | **Interpretation** |
| --- | --- | --- |
| Complete-case event distribution | 2020: 20/28 (71.4%); 2021: 8/28 (28.6%) | Sparse seasonal categories contribute instability. |
| Reference population-average season coefficient | 2.687 (0.358-20.169) | Very wide interval. |
| Reference Firth season coefficient | 2.889 (0.599-14.053) | Very wide interval. |
| PWP gap-time season coefficient | 31.227 (1.874-520.243) | Extremely unstable; season was aliased/dropped in other Cox fits. |
| Firth interaction after removing season | 0.630 (0.444-0.970) | Dropping season did not explain the retrospective interaction. |

**H. Backward-looking expanding-history standardization**

| **Estimator** | **Acute load OR (95% CI)** | **Wellness OR (95% CI)** | **Chronic load OR (95% CI)** | **Previous episode OR (95% CI)** | **Acute x wellness OR (95% CI)** | **N / onsets** |
| --- | --- | --- | --- | --- | --- | --- |
| Firth, team retained | 0.720 (0.392-1.107) | 0.885 (0.526-1.368) | 1.955 (1.208-3.058) | 8.977 (3.417-30.061) | 0.974 (0.728-1.252) | 19,216 / 27 |
| GLMM, team removed | 0.624 (0.347-1.123) | 0.820 (0.480-1.403) | 1.941 (1.162-3.241) | 5.628 (1.014-31.228) | 0.958 (0.697-1.317) | 19,216 / 27 |
| Firth, team removed | 0.676 (0.464-0.961) | 0.893 (0.562-1.363) | 2.068 (1.386-2.908) | 14.510 (5.228-53.279) | 0.944 (0.859-1.137) | 19,216 / 27 |

Alternative-wellness directional checks under backward-looking drop-team Firth showed no inverse interaction signal for either fatigue or readiness; these minimal directional refits are reported without interval estimates because the penalized profile limits were not numerically stable in this specification.

Note. This post-SAP sensitivity used only observations strictly before day t, with at least 10 prior non-missing observations. Because no fixed baseline duration had been specified in the preregistration, expanding history was used for the reported leakage-free sensitivity rather than introducing an additional post hoc fixed-window choice.
