# Supplementary material for: Within-player load-wellness monitoring and lower-limb pain in elite female football: A reproducible secondary analysis of the SoccerMon dataset
Source: PLoS One. 2026 Nov 13;21(11):e0362663. doi: 10.1371/journal.pone.0362663 (PMC13766280; doi:10.1371/journal.pone.0362663)
Supplement: Supplementary file 10 [file pone.0362663.s010.docx]

**S8 Table. Bootstrap and cluster-robust inference.**

| **Estimator** | **Wellness variant** | **Term** | **Median OR** | **Lower 95% limit** | **Upper 95% limit** | **Bootstrap method** |
| --- | --- | --- | --- | --- | --- | --- |
| Marginal IRLS | Soreness | Acute load × soreness burden | 0.670 | 0.447 | 1.107 | Player-cluster percentile bootstrap (B=2,000; 2,000 successful) |
| Firth logistic | Soreness | Intercept | 0.010 | 0.001 | 0.043 | Firth player-cluster percentile bootstrap (B=2,000; 2,000 successful) |
| Firth logistic | Soreness | Acute load (7-day within-player z-score) | 0.610 | 0.250 | 1.101 | Firth player-cluster percentile bootstrap (B=2,000; 2,000 successful) |
| Firth logistic | Soreness | Soreness burden (recent 3-day within-player z-score) | 0.832 | 0.586 | 1.471 | Firth player-cluster percentile bootstrap (B=2,000; 2,000 successful) |
| Firth logistic | Soreness | Acute load × soreness burden | 0.655 | 0.440 | 1.159 | Firth player-cluster percentile bootstrap (B=2,000; 2,000 successful) |
| Firth logistic | Soreness | Chronic load (28-day within-player z-score) | 0.960 | 0.482 | 1.954 | Firth player-cluster percentile bootstrap (B=2,000; 2,000 successful) |
| Firth logistic | Soreness | Previous episode history | 6.263 | 1.891 | 29.068 | Firth player-cluster percentile bootstrap (B=2,000; 2,000 successful) |
| Firth logistic | Soreness | Calendar time | 0.992 | 0.984 | 0.996 | Firth player-cluster percentile bootstrap (B=2,000; 2,000 successful) |
| Firth logistic | Soreness | Team B vs Team A | 0.023 | 0.007 | 0.087 | Firth player-cluster percentile bootstrap (B=2,000; 2,000 successful) |
| Firth logistic | Soreness | 2021 season vs 2020 | 2.914 | 0.346 | 30.762 | Firth player-cluster percentile bootstrap (B=2,000; 2,000 successful) |
| Firth logistic | Fatigue | Intercept | 0.010 | 0.001 | 0.046 | Firth player-cluster percentile bootstrap (B=2,000; 2,000 successful) |
| Firth logistic | Fatigue | Acute load (7-day within-player z-score) | 0.628 | 0.238 | 1.315 | Firth player-cluster percentile bootstrap (B=2,000; 2,000 successful) |
| Firth logistic | Fatigue | Fatigue burden (recent 3-day within-player z-score) | 0.757 | 0.554 | 1.167 | Firth player-cluster percentile bootstrap (B=2,000; 2,000 successful) |
| Firth logistic | Fatigue | Acute load × fatigue burden | 0.867 | 0.618 | 1.249 | Firth player-cluster percentile bootstrap (B=2,000; 2,000 successful) |
| Firth logistic | Fatigue | Chronic load (28-day within-player z-score) | 0.973 | 0.505 | 1.928 | Firth player-cluster percentile bootstrap (B=2,000; 2,000 successful) |
| Firth logistic | Fatigue | Previous episode history | 5.906 | 1.555 | 31.800 | Firth player-cluster percentile bootstrap (B=2,000; 2,000 successful) |
| Firth logistic | Fatigue | Calendar time | 0.992 | 0.984 | 0.996 | Firth player-cluster percentile bootstrap (B=2,000; 2,000 successful) |
| Firth logistic | Fatigue | Team B vs Team A | 0.026 | 0.010 | 0.096 | Firth player-cluster percentile bootstrap (B=2,000; 2,000 successful) |
| Firth logistic | Fatigue | 2021 season vs 2020 | 2.728 | 0.364 | 41.510 | Firth player-cluster percentile bootstrap (B=2,000; 2,000 successful) |
| Firth logistic | Readiness | Intercept | 0.010 | 0.001 | 0.047 | Firth player-cluster percentile bootstrap (B=2,000; 1,999 successful; 1 failed) |
| Firth logistic | Readiness | Acute load (7-day within-player z-score) | 0.676 | 0.281 | 1.259 | Firth player-cluster percentile bootstrap (B=2,000; 1,999 successful; 1 failed) |
| Firth logistic | Readiness | Readiness deficit (recent 3-day within-player z-score) | 1.026 | 0.855 | 1.227 | Firth player-cluster percentile bootstrap (B=2,000; 1,999 successful; 1 failed) |
| Firth logistic | Readiness | Acute load × readiness deficit | 0.885 | 0.621 | 1.252 | Firth player-cluster percentile bootstrap (B=2,000; 1,999 successful; 1 failed) |
| Firth logistic | Readiness | Chronic load (28-day within-player z-score) | 1.014 | 0.522 | 2.024 | Firth player-cluster percentile bootstrap (B=2,000; 1,999 successful; 1 failed) |
| Firth logistic | Readiness | Previous episode history | 6.211 | 1.745 | 28.663 | Firth player-cluster percentile bootstrap (B=2,000; 1,999 successful; 1 failed) |
| Firth logistic | Readiness | Calendar time | 0.992 | 0.984 | 0.996 | Firth player-cluster percentile bootstrap (B=2,000; 1,999 successful; 1 failed) |
| Firth logistic | Readiness | Team B vs Team A | 0.026 | 0.009 | 0.102 | Firth player-cluster percentile bootstrap (B=2,000; 1,999 successful; 1 failed) |
| Firth logistic | Readiness | 2021 season vs 2020 | 2.496 | 0.332 | 39.725 | Firth player-cluster percentile bootstrap (B=2,000; 1,999 successful; 1 failed) |
| Marginal IRLS (drop team) | Soreness | Acute load x soreness burden | 0.740 | 0.556 | 1.132 | Player-cluster percentile bootstrap (B=2,000; 2,000 successful; P[OR>1]=0.102) |
| Marginal IRLS (non-overlap + drop team) | Soreness | Acute load x soreness burden | 0.740 | 0.557 | 1.133 | Player-cluster percentile bootstrap (B=2,000; 2,000 successful; P[OR>1]=0.102) |

Note. ORs and 95% limits are percentile summaries on the odds-ratio scale. Firth estimates use player-cluster bootstrap replicates; the readiness model had one failed replicate. Team coefficients are not substantively interpreted because the complete-case sample contains no Team B onset. The final two rows are post-SAP bootstrap sensitivities.
