# Supplementary material for: Within-player load-wellness monitoring and lower-limb pain in elite female football: A reproducible secondary analysis of the SoccerMon dataset
Source: PLoS One. 2026 Nov 13;21(11):e0362663. doi: 10.1371/journal.pone.0362663 (PMC13766280; doi:10.1371/journal.pone.0362663)
Supplement: Supplementary file 11 [file pone.0362663.s011.docx]

**S9 Table. Missing-data and inverse-probability-weighting analyses.**

**A. Raw day-level wellness reporting missingness**

| **Wellness** | **Missingness: full 36,550-day grid** | **Observed / total: full grid** | **Missingness: monitoring window** | **Observed / total: monitoring window** |
| --- | --- | --- | --- | --- |
| Fatigue | 53.5% | 16,992 / 36,550 | 32.0% | 16,992 / 25,003 |
| Readiness | 53.5% | 16,997 / 36,550 | 32.0% | 16,997 / 25,003 |
| Soreness | 53.5% | 16,999 / 36,550 | 32.0% | 16,999 / 25,003 |

**B. Final complete-case model availability**

| **Wellness** | **Risk-set rows** | **Complete-case N (%)** | **Players** | **Onset-days** | **Event players** |
| --- | --- | --- | --- | --- | --- |
| Soreness | 36,296 | 19,758 (54.4%) | 49 | 28 | 9 |
| Fatigue | 36,296 | 19,815 (54.6%) | 50 | 28 | 9 |
| Readiness | 36,296 | 19,817 (54.6%) | 50 | 28 | 9 |

**C. Observation-model specification**

| **Wellness** | **Observation-model variant** | **AUC** | **Analysis timing** | **Specification difference** |
| --- | --- | --- | --- | --- |
| Soreness | Includes onset indicator | 0.932 | Revision-stage SAP | daily_load_z + team + season + calendar_time_z + day of week + lag-1 reporting indicator + previous episode history + onset_day |
| Soreness | Omits onset indicator | 0.932 | Post-SAP sensitivity | Same observation model, but onset_day omitted |
| Fatigue | Includes onset indicator | 0.932 | Revision-stage SAP | daily_load_z + team + season + calendar_time_z + day of week + lag-1 reporting indicator + previous episode history + onset_day |
| Fatigue | Omits onset indicator | 0.932 | Post-SAP sensitivity | Same observation model, but onset_day omitted |
| Readiness | Includes onset indicator | 0.932 | Revision-stage SAP | daily_load_z + team + season + calendar_time_z + day of week + lag-1 reporting indicator + previous episode history + onset_day |
| Readiness | Omits onset indicator | 0.932 | Post-SAP sensitivity | Same observation model, but onset_day omitted |

**D. Stabilized-weight diagnostics**

| **Wellness** | **Obs. model** | **Max untruncated weight** | **1st / 99th percentile** | **Max truncated weight** | **ESS in CC sample** | **ESS / CC N** |
| --- | --- | --- | --- | --- | --- | --- |
| Soreness | Includes onset | 11.25 | 0.465 / 10.665 | 10.67 | 7,107 | 36.0% |
| Soreness | Omits onset | 11.18 | 0.465 / 10.621 | 10.62 | 7,115 | 36.0% |
| Fatigue | Includes onset | 11.26 | 0.465 / 10.672 | 10.67 | 7,079 | 35.7% |
| Fatigue | Omits onset | 11.19 | 0.465 / 10.629 | 10.63 | 7,086 | 35.8% |
| Readiness | Includes onset | 11.24 | 0.465 / 10.625 | 10.62 | 7,059 | 35.6% |
| Readiness | Omits onset | 11.18 | 0.465 / 10.593 | 10.59 | 7,067 | 35.7% |

**E. Recent-3-day / lag-day alignment audit**

| **Wellness** | **Complete-case N** | **t-1 raw wellness observed** | **t-1 raw wellness missing** | **Interpretation** |
| --- | --- | --- | --- | --- |
| Soreness | 19,758 | 84.2% | 3,112 (15.8%) | Recent-3-day exposure may come from t-2 or t-3 while the observation model's lag-day reporting indicator refers to t-1. |
| Fatigue | 19,815 | 84.1% | 3,151 (15.9%) | Recent-3-day exposure may come from t-2 or t-3 while the observation model's lag-day reporting indicator refers to t-1. |
| Readiness | 19,817 | 84.1% | 3,148 (15.9%) | Recent-3-day exposure may come from t-2 or t-3 while the observation model's lag-day reporting indicator refers to t-1. |

**F. Final weighted interaction estimates**

| **Wellness** | **Observation model** | **N / onsets** | **Acute load x wellness OR (95% CI)** | **p** | **Analysis timing** |
| --- | --- | --- | --- | --- | --- |
| Soreness | Includes onset | 19,758 / 28 | 0.646 (0.429-0.973) | 0.037 | Revision-stage SAP |
| Soreness | Omits onset | 19,758 / 28 | 0.657 (0.425-1.015) | 0.059 | Post-SAP sensitivity |
| Fatigue | Includes onset | 19,815 / 28 | 0.801 (0.616-1.042) | 0.098 | Revision-stage SAP |
| Fatigue | Omits onset | 19,815 / 28 | 0.789 (0.612-1.016) | 0.066 | Post-SAP sensitivity |
| Readiness | Includes onset | 19,817 / 28 | 0.918 (0.695-1.213) | 0.549 | Revision-stage SAP |
| Readiness | Omits onset | 19,817 / 28 | 0.901 (0.684-1.188) | 0.461 | Post-SAP sensitivity |

Notes. AUC, area under the receiver-operating-characteristic curve; CC, complete case; CR2, small-sample bias-reduced cluster-robust covariance estimator; ESS, effective sample size; IPW, inverse-probability weighting; OR, odds ratio. Stabilized weights were truncated at the 1st and 99th percentiles. The weighted models are sensitivity analyses under strong observation-model and positivity assumptions; the approximately 36% CC-sample ESS and the lag-alignment limitation mean that IPW cannot be interpreted as eliminating selection or missing-data bias.
