# Supplementary material for: Within-player load-wellness monitoring and lower-limb pain in elite female football: A reproducible secondary analysis of the SoccerMon dataset
Source: PLoS One. 2026 Nov 13;21(11):e0362663. doi: 10.1371/journal.pone.0362663 (PMC13766280; doi:10.1371/journal.pone.0362663)
Supplement: Supplementary file 12 [file pone.0362663.s012.docx]

**S10 Table. Protocol and analysis-plan reconciliation.**

| **Item** | **Original OSF preregistration (29 May 2026)** | **Frozen revision-stage SAP (22 July 2026) / implemented reference analysis** | **Later revision-stage analyses / final status** |
| --- | --- | --- | --- |
| Primary outcome | New onset on day t of a self-reported lower-limb injury/pain episode; 7-day washout. | Returned to the registered day-t onset estimand on at-risk player-days; same-day multi-location onsets collapsed. | No further estimand change. Final manuscript consistently describes self-reported symptom episodes, not clinician-confirmed diagnoses. |
| Outcome timing / risk set | Days during an ongoing episode are not at risk for a new episode; lagged exposures precede day t. | Explicit recurrent-event risk set: ongoing-episode days excluded; recovery at end+1; end+1+washout retained as sensitivity. | No later change to the reference risk set; event concentration was quantified at episode, risk-set onset-day, and complete-case onset-day levels. |
| Observation period | Each player's observation period begins at the first valid subjective monitoring record and ends at the last valid subjective monitoring record. | The implemented reference analysis used a common 1 Jan 2020-31 Dec 2021 calendar grid; model-specific complete cases excluded rows without required monitoring variables. This differed from the registered player-specific observation period. | Protocol deviation retained and explicitly disclosed in Methods and this table. |
| Acute load | 7-day rolling mean over t-7:t-1, excluding outcome day. | Implemented as lagged acute load and within-player standardized. | No later change to the acute window. |
| Chronic load | 28-day rolling mean over t-28:t-1, excluding outcome day. | Implemented as lagged 28-day chronic load and within-player standardized. | Post-SAP sensitivity used a non-overlapping t-28:t-8 chronic window; this was not part of the original preregistration. |
| Within-player standardization | Exposures centered on each player's own mean and, where appropriate, divided by that player's own SD; no prospective baseline rule was specified. | Reference reanalysis used full-period within-player mean/SD (retrospective parameterization). | Post-SAP sensitivity recalculated z-scores from expanding history strictly before day t (minimum 10 prior observations), eliminating future-data leakage without introducing a post hoc fixed-duration window. |
| Wellness polarity | Soreness/fatigue coded so higher transformed values are worse; readiness reverse-coded so higher transformed values are worse. | Canonical coding: fatigue_burden=6-fatigue; soreness_burden=6-soreness; readiness_deficit=11-readiness. | Soreness polarity provenance was independently verified from PmSys/Jarmann documentation without using onset status, outcome associations, coefficients, p values, or outcome correlations. |
| Focal interaction hierarchy | Primary interaction: acute load x soreness; fatigue/readiness are alternative wellness interactions. | The focal acute-load x soreness interaction was retained. The frozen SAP designated a player-random-intercept logistic mixed model (Model A) as primary and Firth logistic regression (Model F) as a small-sample companion. | Final reporting emphasized Firth and whole-player bootstrap uncertainty because of sparse complete-case events and team separation. The focal interaction hypothesis was not changed. |
| Wellness model structure | Main-effects model planned soreness, fatigue, and readiness jointly; acute load x soreness was the focal interaction, with fatigue/readiness replacement interactions specified as sensitivity analyses. | Wellness variants were fitted in separate model families; soreness remained the focal variant and fatigue/readiness were evaluated as alternative wellness variants. | No new focal wellness hypothesis was introduced. |
| Firth specification / player dependence | The original preregistration planned recurrent-onset modeling with player-level dependence through a random intercept; Firth was not the preregistered primary estimator. | The frozen SAP listed Firth (Model F) as a sparse-data companion to the intended primary GLMM (Model A). The implemented Firth model (R logistf) included acute load x soreness, chronic load, previous-episode history, calendar time, team and season; it did not contain a player random intercept or cluster-robust SE. | Firth profile-likelihood CIs were model-based, not player-cluster adjusted. Separately, 2,000 whole-player bootstrap refits provided percentile CIs (S8 Table). With only nine complete-case event players, this assessment remains imprecise. |
| Previous episode history | Previous injury history or recent lower-limb episode status where estimable. | Previous-episode history included in the reference models. | No later definitional change; final interpretation emphasizes that the estimate is large but imprecise and not a validated prediction rule. |
| Covariates | Player random intercept; team, season/season phase, calendar time; add only if stable. | The SAP Model A included a player random intercept, event-history terms, calendar time, team and season; in the implemented Firth model, the same substantive covariates entered as fixed effects without a player random intercept. | Drop-team and drop-season analyses were added after SAP freeze to quantify separation/instability; these are post-SAP sensitivity analyses, not originally prespecified analyses. |
| Recurrent-event handling | Player random intercept and at-risk recurrent-onset framework; details limited. | SAP candidates included the player-random-intercept discrete-time GLMM; Andersen-Gill/PWP Cox with player-cluster robust SE; shared-frailty Cox; and Firth with separate whole-player bootstrap inference. | Post-SAP work added targeted exposure, team and season sensitivities; it did not turn the Firth model-based confidence intervals into cluster-adjusted intervals. |
| Missing data / IPW | Model-specific complete cases; report missingness. Conservative zero-vs-missing training-day sensitivity planned. No primary multiple imputation. | Complete-case reference plus stabilized IPW sensitivity with 1st/99th percentile truncation; IPW never interpreted as eliminating bias. | A post-SAP observation-model variant omitted onset_day. Final reporting includes ESS, positivity, weight tails, and lag-alignment limitations. |
| Unreported zero-load sensitivity | Treat potentially unreported training-load days as missing rather than zero when monitoring context does not support a rest-day interpretation. | Not implemented because the available data do not reliably distinguish true rest days from unreported training days without a post hoc classification rule. | Non-implementation is explicitly disclosed as a protocol deviation; exposure-misclassification direction remains uncertain. |
| Sensitivity analyses | 3/14-day washout, alternative training-load metrics/windows, wellness swap, risk-set rule, conservative missingness sensitivity; knee/major analyses if counts permit. | Implemented 3/14-day washout, risk-set re-entry, ACWR, strict t-1 wellness, highest-contributor exclusion; old future-risk-window analysis demoted. | Added after SAP freeze: non-overlap chronic window, drop team, drop season, backward-looking standardization, IPW no-outcome variant, event-concentration audit, and model-space census. These are not described as originally prespecified. |
| Team / season instability | Team and season allowed as covariates, conditional on stability. | Team B had no complete-case onset event, producing complete separation; season coefficient was wide/aliased in some estimators. | Post-SAP drop-team/drop-season checks showed that removing these covariates did not explain the retrospective focal interaction; imbalance remains a limitation on precision/generalization. |
| Multiplicity | Primary/alternative/exploratory hierarchy specified; no detailed model-space census. | Revision-stage SAP emphasized one focal interaction and secondary/sensitivity labeling; no new multiplicity correction was introduced. | Final scientifically reported space: 49 successfully fitted specifications, 147 focal tests, including 30 interaction tests; internal QA, failed fits, and rejected fallback levels excluded from this denominator. Full computational registry remains archived. |
| Interpretation scope | Explanatory, not predictive; self-reported injury/pain episodes; no clinical diagnosis, causal claim, or actionable threshold. | Same scope retained. | Final manuscript further limits implementation claims because full-period z-scores are retrospective and the backward-looking sensitivity materially changed the focal interaction and chronic-load estimate. |
