# Supplementary material for: Within-player load-wellness monitoring and lower-limb pain in elite female football: A reproducible secondary analysis of the SoccerMon dataset
Source: PLoS One. 2026 Nov 13;21(11):e0362663. doi: 10.1371/journal.pone.0362663 (PMC13766280; doi:10.1371/journal.pone.0362663)
Supplement: Supplementary file 13 [file pone.0362663.s013.docx]

S1 File. Code reproduction record

**Within-player load–wellness monitoring and lower-limb pain in elite female football: a reproducible secondary analysis of the SoccerMon dataset**

*PONE-D-26-31950*

# Scope and reference analysis

This record describes the accompanying S2 File reproducibility archive for the revised analysis. The S2 archive was assembled from the final R2 research freeze after analysis completion. Archive assembly did not refit models, rerun bootstrap procedures, or alter inferential estimates.

The locked R1 reference run is RUN_20260722_151418 and was reproduced as RUN_R2_REPRO. The source R2 freeze records FINAL ANALYSIS FREEZE: PASS. Its complete research-freeze hash is 5e2522a5b5bbbb4df370b0bdffcfa4747363d03a253d43797ec2c295e75421e9.

The public SoccerMon raw archive is not redistributed in S2 File. The required input is subjective.zip from Zenodo (DOI: 10.5281/zenodo.10033832); the expected SHA-256 record is included in 00_INPUT_SNAPSHOT/subjective.zip.sha256.

The primary risk set contains 36,296 player-days, 42 lower-limb onset-days, and 15 event players. The principal complete-case wellness models contain 28 onset-days from 9 event players. These event counts are the binding information limit for the inferential analyses.

# Freeze verification

Gate A (R1 reproduction): 217 checks, 0 FAIL. Numeric comparisons of the reproduced reference outputs were within the prespecified tolerances, with exact or normalized agreement where applicable.

Gate B (independent raw rebuild): 33/33 checks MATCH, with maximum absolute numerical difference 0.0. The raw rebuild recovered the 50-player × 731-day grid, the lower-limb episode inventory, onset-day set, and primary modelling dataset used by the reference analysis.

Final cross-file validation: 54/54 numeric-consistency checks PASS. The final master results contain 678 rows with no duplicate keys, and the R2 model sets reconcile with the model registry.

The independent wellness-polarity audit classified all seven audited codings as CONSISTENT with HIGH confidence and recorded that outcome data were not used for the coding assessment.

# R2 analyses retained in the archive

The analysis taxonomy distinguishes R2_REVIEWER_REQUESTED analyses from R2_INTERNAL_QA_ONLY analyses. Reviewer-requested workstreams include acute/chronic collinearity diagnostics, a non-overlapping chronic-load window, drop-team sensitivity, season sensitivity, backward-looking within-player standardization, the independent polarity audit, the final IPW analysis, event-influence analyses, and the model-space/multiplicity census.

The final IPW workstream preserves both observation-model specifications. For the acute-load × soreness interaction, the with-onset observation model gives OR 0.6464 (95% CI 0.4293–0.9732), whereas the no-outcome observation model gives OR 0.6567 (0.4247–1.0155). Effective sample sizes are approximately 36% of the corresponding complete-case sample, so the IPW analyses are retained as sensitivity analyses rather than treated as definitive correction for missingness.

The material R2 sensitivity finding is the backward-looking expanding-history standardization: the soreness interaction attenuates to approximately OR 0.94–0.98 across estimators, while the chronic-load coefficient changes direction (approximately OR 1.94–2.14). The fixed 90-day rolling-baseline analysis is retained only as internal QA.

# S2 File organization

| **Directory** | **Contents** |
| --- | --- |
| 00_INPUT_SNAPSHOT | Source and raw-input hash records; raw ZIP not redistributed. |
| 01_ENVIRONMENT | Python/R package and session records with workstation-specific paths neutralized. |
| 02_R1_REPRODUCTION | R1 analysis code, core derived inputs, and RUN_R2_REPRO outputs used by the R2 workstreams. |
| 03_RAW_REBUILD | Selected raw-rebuild audit outputs supporting Gate B. |
| 04–17 | R2 taxonomy, audits, sensitivity analyses, model registry, focal-stability summary, and MASTER_RESULTS_R2. |
| 18_LOGS | Frozen execution logs required for computational traceability. |
| 19_VALIDATION | Gate A, Gate B, and 54-check final validation records. |
| 20_FREEZE | Final freeze status, source freeze hash, final report, response map, and freeze-building scripts. |
| 99_ARCHIVE_INTEGRITY | Member SHA-256 manifest and source-to-archive mapping for the distributed ZIP. |

# Reproduction procedure

Extract S2_File.zip to a local working directory. The R2 workstream scripts are preserved as executed in the final freeze. Several scripts define the original freeze root through an FR (or equivalent) constant. On another computer, replace only that root value with the extracted S2 directory. This is a path relocation only; the analytical specifications and transformations should not be changed.

The included 02_R1_REPRODUCTION/r1_sandbox/07_ANALYSIS_OUTPUTS/modeling_dataset.csv and 02_R1_REPRODUCTION/r1_sandbox/02_RAW_DATA/rebuilt_player_day_base.csv provide the core derived inputs used by the R2 workstreams. The RUN_R2_REPRO directory preserves the reproduced R1 reference outputs and IPW weight files required by later sensitivity analyses.

For an independent raw-data rebuild, obtain subjective.zip from the cited Zenodo record, verify its hash against 00_INPUT_SNAPSHOT/subjective.zip.sha256, and use the raw-audit/rebuild code retained under 02_R1_REPRODUCTION/r1_sandbox/06_ANALYSIS_CODE. Gate B outputs in 03_RAW_REBUILD and 19_VALIDATION provide the reference reconciliation targets.

Random procedures used seed 20260722; bootstrap procedures requested B=2000 unless otherwise specified in the individual workstream files. Failed or non-estimable models are retained in the diagnostics and model registry rather than silently omitted.

# Archive scope and integrity

The distributed S2 File is intentionally smaller than the private research freeze. It omits the machine-specific installed R library/cache, the separately available public raw ZIP, duplicated historical submission packages, third-party provenance cache documents/screenshots, and selected regenerable intermediate sensitivity datasets. The omission policy is documented in 00_ARCHIVE_SCOPE/ARCHIVE_SCOPE.md.

The complete private freeze remains identified by 20_FREEZE/FREEZE_HASH.txt. The distributed archive has its own member-level integrity manifest in 99_ARCHIVE_INTEGRITY/archive_sha256_manifest.csv and a source-to-archive mapping in source_to_archive_map.csv.

S2_File.zip SHA-256: d9c9d3cbfa31bd56717ff5c3c925ebe8f74c14062498f0f4ab0bda9d65594577.

# Interpretive limits

The final freeze should be interpreted within the sparse-event setting. Multiple model specifications do not increase the underlying outcome information beyond the observed onset-days and event players. The archive therefore supports the manuscript wording that there is no stable evidence of association across the complete sensitivity set; it does not support interpreting predominantly null estimates as evidence of no association.

Shared-frailty results are retained for diagnostic transparency. Frailty variance was not estimable for the soreness and fatigue fits, and the readiness frailty fit failed. These fits are not used as corroborating evidence.
